# Preparation, Characterization, and Application of Metal Oxide-Doped Zeolitic Imidazolate Framework

**DOI:** 10.1021/acsomega.3c03509

**Published:** 2023-07-20

**Authors:** Fulya Kümbetlioğlu, Kürşad Oğuz Oskay, Zafer Çıplak, Ayten Ateş

**Affiliations:** † Faculty of Engineering, Department of Chemical Engineering, Sivas Cumhuriyet University, Sivas 58140, Turkey; ‡ Faculty of Engineering, Department of Metallurgical and Materials Engineering, Sivas Cumhuriyet University, Sivas 58140, Turkey

## Abstract

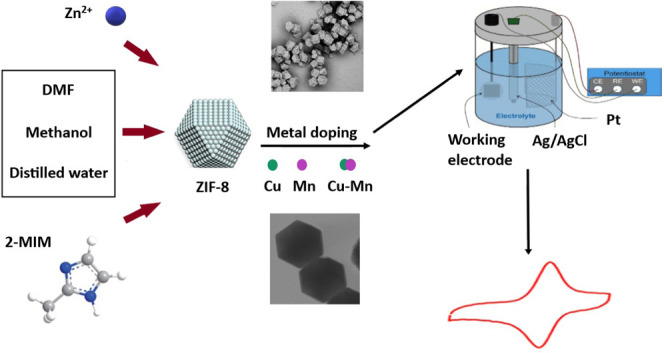

Metal–organic frameworks (MOFs) attract the attention of researchers due to their unique properties, such as high surface area, porosity, and stability. Therefore, in this study, the synthesis of zeolitic imidazole frameworks (ZIF-8), a subclass of MOFs, and copper oxide (Cu_2_O) and manganese oxide (MnO_2_) containing ZIF-8 was carried out by a mixing method with methanol. The characterization results show that the polyhedral structure of ZIF-8 was prepared with a surface area of 2088 m^2^/g and a crystallite size of 43.48 nm. Then, each and mixture of two metal oxides were introduced into the ZIF-8 crystal structure. It was found that the surface area and pore volumes of all metal/ZIF-8 samples decreased with metal loading, depending on the type and ratio of metal oxides. The ZIF-8 containing 4.0 wt % Cu_2_O and 1.0 wt % MnO_2_ had the highest surface area (2084 m^2^/g), which was closest to that of ZIF-8. The polyhedral structure was maintained by the addition of both metal oxides, and the crystal size of the material decreased with the loading of MnO_2_ to the ZIF-8 structure. All of the synthesized samples were analyzed in supercapacitor applications and a relatively higher value of specific capacitance was obtained for Cu–Mn/ZIF-8 due to higher surface area and improved conductivity. In addition to supercapacitor applications, the properties of metal/ZIF-8 are also promising for applications such as catalysts, membranes, and gas storage.

## 1 Introduction

Metal–organic frameworks (MOFs) are porous compounds made of a metal supply and an organic ligand. Transition metals such as Co^2+^ and Zn^2+^ are used to obtain these MOF crystals. Organic binders, on the other hand, are usually composed of long-chain alkyl or aryl groups and are another factor in the formation of the MOF structure.^1^ In contrast to conventional microporous and mesoporous materials, MOFs have uniformly sized pores and extremely high surface area. Due to these advantages, MOFs have recently been used in various fields such as gas sorption/separation, energy storage systems, and catalysis.^2^

Zeolitic imidazole frameworks (ZIF-8), a subclass of MOFs, consist of tetrahedrally coordinated zinc ions bonded with 2-methylimidazole, resulting in a sodalite-zeolite structure. ZIF-8 attracts attention because it has advantages such as a large surface area, controllable pore size, and high stability.^3^ The synthesis method, solvent, and concentrations of reactants can affect the properties of ZIF-8. There are three main methods for the synthesis of ZIF-8: mixing methods, solvothermal methods, and sonochemical methods. The production yield is influenced by the different solvents used in ZIF-8 synthesis, including dimethylformamide (DMF), distilled water, and methanol.^4^ Therefore, the choice of the right solvent and synthesis technique is crucial for the creation of a ZIF-8 structure. When methanol is employed as the solvent, molecular interactions of reactants with the solvent that have the capacity to transmit hydrogen bonds, increase the pace of the synthesis reaction, aid in the deprotonation of the ligand, and make it easier for the ligand to coordinate with Zn^2+^.^5^ Depending on the structure of ZIF-8, they are used in various fields, such as catalysts,^6^ adsorbents,^7^ energy storage systems,^8^ and membranes.^9^ Membranes built on ZIF-8 also have many potential applications in nanofiltration. Wang et al. introduced ZIF-8 nanoparticles simultaneously into alumina pores using a freeze-assisted counter-diffusion approach.^9^ They found that the resulting membrane exhibited an acceptable salt permeability for antimicrobial desalination and good pharmacological rejection. In addition, ZIF-8 was experimentally investigated as anodes for Na^+^ and K^+^ batteries by Yu et al. Their findings demonstrated that zinc sites, particularly when the initial atom is introduced, have large adsorption energies for alkali metal atoms.^10^ Although ZIF-8 has advantages for these applications, the weak properties of pure ZIF-8, such as low conductivity, low activity, poor reusability, etc., need to be improved in catalytic applications. It is reported in the literature that a number of metals improve catalytic activity and conductivity.^3^ For example, cobalt and cerium catalysts have high activity and stability.^11^ In addition, iron, chromium, titanium, and manganese play important roles in catalytic reactions and have high affinity for hydrogen storage.^12^ Moreover, ZIF-8 with transition-metal oxides has been applied in a variety of industries. Li and colleagues discovered that the Cu_2_O@ZIF-8 nanocomposite was effective at hydrogenating 4-nitrophenol and had high cyclic stability.^13^ They reported that the protected lattice structure of ZIF-8 accelerated the process by which bare Cu_2_O began to decompose rapidly. Energy storage systems are another application. Composites of ZIF-8 with Fe_2_O_3_, MnO_2_, ZnO, and Co_3_O_4_ are widely used electrode materials to increase the specific capacitance in electrochemical energy storage systems. Additionally, compared to carbon-based materials, transition-metal oxides provide greater energy densities.^14^ The electrochemical performance of the solvothermal synthesized Fe_3_O_4_/ZIF-8 and Fe_3_O_4_/ZIF-67 materials was examined in the literature.^15^ Due to the maximal use of the active material, the porous structure of the zeolitic imidazole framework combined with Fe_3_O_4_ offers benefits including a short ion diffusion route, quick ion/electron transfer, and high specific capacitance.^15^

For the above reasons and because there are only few studies in the literature on binary metals containing ZIF-8, in this study, the synthesis of ZIF-8 and metal oxides (Cu_2_O and MnO_2_) containing ZIF-8 was carried out by a mixing method with methanol. The samples were characterized by scanning electron microscopy (SEM), scanning transmission electron microscopy (STEM), X-ray diffraction (XRD), X-ray photoelectron spectroscopy (XPS), N_2_ adsorption–desorption, and Fourier transform infrared (FTIR). Their electrochemical performances were analyzed with cyclic voltammetry measurements in various potential windows and at different scan rates. The electrochemical performances were interpreted from the results as a function of species and different metal ratios.

## 2 Materials and Methods

### 2.1 Chemicals

Zinc nitrate hexahydrate (98%), 2-methylimidazole (99%), ethanol (≥99.8%), methanol (≥99.9%), Nafion (5 wt %), and copper(I) oxide (97%) were purchased from Sigma-Aldrich. Manganese(IV) oxide (>90%), *N*,*N*-dimethylformamide, and sodium sulfate were purchased from Merck.

#### 2.1.1 ZIF-8 Synthesis

The ZIF-8 samples were prepared using a mixing method with three different solvents.^4^ The details of the synthesis method are explained below.

##### 2.1.1.1 Preparation with Methanol

The synthesis of ZIF-8 was carried out using methanol as a solvent. For this purpose, 5.95 g of Zn(NO_3_)_2_·6H_2_O and 6.57 g of 2-methylimidazole (2-MIM) were dissolved separately in 100 mL of anhydrous methanol and mixed with a magnetic stirrer at 200 rpm for 5 min.^16^ The two solutions were placed together in a flask and stirred for 1 h at 25 °C. The solution was washed three times with methanol and then centrifuged at 5000 rpm for 5 min. The solid was dried overnight at 60 °C. This sample was designated as ZIF-8/1. The solution was dried in a freeze drier (Labconco) for 12 h to analyze the impact of drying on the structure of ZIF-8. This sample was designated as ZIF-8/2.

##### 2.1.1.2 Preparation with Dimethylformamide

For the synthesis of ZIF-8 with DMF, 3 g of Zn(NO_3_)_2_·6H_2_O and 6.6 g of 2-MIM were dissolved separately in 50 mL of DMF and mixed for 5 min.^4^ The solutions were then mixed for another 6 h at room temperature in a magnetic stirrer, washed four times with methanol, and centrifuged at 5000 rpm for 5 min. ZIF-8 was dried in an oven at 60 °C for 12 h. This sample was named ZIF-8/3.

##### 2.1.1.3 Preparation with Distilled Water

For the synthesis of ZIF-8 with distilled water, 1.17 g of Zn(NO_3_)_2_·6H_2_O was quickly added to a solution of 8 mL of distilled water. 22.7 g of 2-MIM was dissolved in 80 mL of distilled water and mixed for 5 min.^17^ The two solutions were mixed for 1 h at 200 rpm by using a magnetic stirrer. With distilled water and ethanol, the solution was centrifuged four times in succession. A vacuum oven was then used to dry the sample. This sample was named ZIF-8/4.

#### 2.1.2 Preparation of Metal/ZIF-8

Manganese (Mn) and copper (Cu) oxides, and their combinations were added into the ZIF-8 structure. Cu_2_O and MnO_2_ were used as metal sources. To protect the structure of ZIF-8 with metal loading, the molar ratio of metal oxide and zinc nitrate hexahydrate was kept at 5%.^3^

First, 20 mL of methanol was used to dissolve 2.125 mmol of Zn(NO_3_)_2_·6H_2_O and 0.125 mmol of metal oxide in order to create metal/ZIF-8 nanocomposites.^18^ 3.28 g of 2-MIM was dissolved simultaneously in an 80 mL solution of methanol. The solutions were completely blended for 2 h at 200 rpm while at room temperature. The process used to create the metal/ZIF-8 nanocomposites included washing three times with methanol, centrifugation, and overnight drying.

##### 2.1.2.1 Preparation of Cu–Mn/ZIF-8 in Different Mole Amounts

In the synthesis of Cu–Mn/ZIF-8s, the mole percent ratios of copper and manganese oxide were 4:1, 2.5:2.5 (R1), 1:4 (R2), and 2:3 (R3), respectively. The same procedure was used as for the metal/ZIF-8 synthesis method.

#### 2.1.3 Characterization

To examine the morphology and element composition of ZIF-8 and metal/ZIF-8 samples, a scanning transmission electron microscope (STEM) and a field emission scanning electron microscope (FESEM) were utilized. For this reason, FESEM was made by Tescan Mira3 XMU (Brno, Czech Republic), and equipped EDS (Oxford Inst. INCA) was used to investigate the particles and determine the element composition. In addition, the metal content of samples was determined by X-ray fluorescence (XRF) (Thermo Scientific Niton XL3t Goldd+). By measurement of the diameter of the particles, which were then represented in STEM images using the Microsoft JMicroVision application, the particle size distribution was ascertained. The X-ray diffraction (XRD) method was used to determine the crystallographic properties of the samples and the phases they contained. A Bruker D8 Advance X-ray diffractometer with a D/tex Ultra 250 detector and copper (Cu) Kα (λ = 1.5 Å) light at 45 kW and 40 mA was used for XRD analysis. The crystallite sizes of ZIF-8 and metal/ZIF-8 samples were determined with the Scherrer equation using Origin 2019b. A FTIR spectrophotometer called Spectrum One FTIR (BrukerAplha) was used to record Fourier transform infrared (FTIR) spectra. Using a Quantachrome 1 C instrument, the samples’ specific surface area and pore volume were calculated using the Brunauer–Emmett–Teller (BET) and Barrett–Joyner–Halenda (BJH) techniques, respectively. Utilizing an X-ray photoelectron spectrometer (XPS) from Thermo Scientific/K-Alpha Brand, chemical constituents and valence states were investigated.

#### 2.1.4 Electrochemical Measurements

The Gamry Instrument Potentiostat/Galvanostat/ZRA in a three-electrode configuration was used to conduct electrochemical experiments. The working electrodes were prepared from the synthesized composites. 2 mg of the synthesized active material, 15 μL of Nafion, and 1.5 mL of ethanol were homogenized in the ultrasonic system for 30 min to prepare the electrode. The solution was then applied to the glassy carbon electrode (GCE), which was subsequently dried in a 50 °C oven. As the counter electrode and reference electrode, Ag/AgCl and Pt plates were employed, respectively. Cyclic voltammetry (CV) was used to determine the specific capacitance of electrodes in different potential windows (−1.2 to −0.2 V, −1.0 to 0 V, and −0.8 to 0.2 V) and at different scan rates (5–200 mV/s). All electrochemical measurements were performed in a 0.1 M Na_2_SO_4_ electrolyte solution. Following the cyclic voltammograms, the specific capacitance values for each potential range were determined using eq 1.^19^
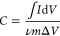
1

where *C* is the capacitance (F/g), Δ*V* is the voltage widow (V), ν is the scan rate (mV/s), *m* is the mass of electrode material (g), and *I* is the response current (A).

## 3 Results and Discussion

### 3.1 Characterization

Table S1 shows the elemental compositions of the ZIF-8 and metal/ZIF-8 samples. ZIF-8 consists of 41.61% C, 22.59% N, 1.0% O, and 34.47% Zn. After Cu_2_O doping, the oxygen content of the material increased from 1.0 to 9.35% in the presence of Cu_2_O oxides, and a loading of 17.46% Cu in ZIF-8 was determined. Similarly, MnO_2_ doping increased the oxygen content, retaining most of the MnO_2_, and 12.71% of Mn was introduced into the structure. The addition of a Cu_2_O and MnO_2_ mixture into the ZIF-8 preparation solution decreased the loading of metal oxides into the ZIF-8 structure, which could be due to the decreasing solubility in the presence of both metal oxides.

**Figure 1:**
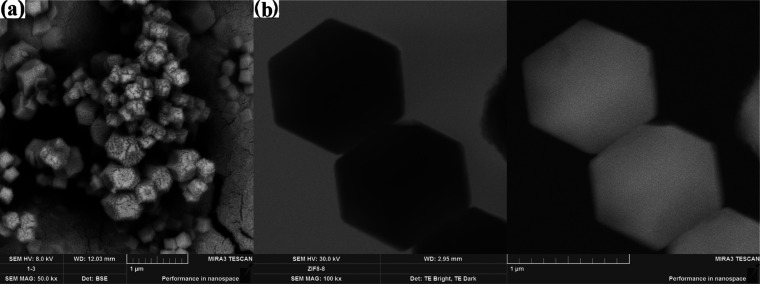
SEM (a) and STEM (b) images of ZIF-8/1.

**Figure 2:**
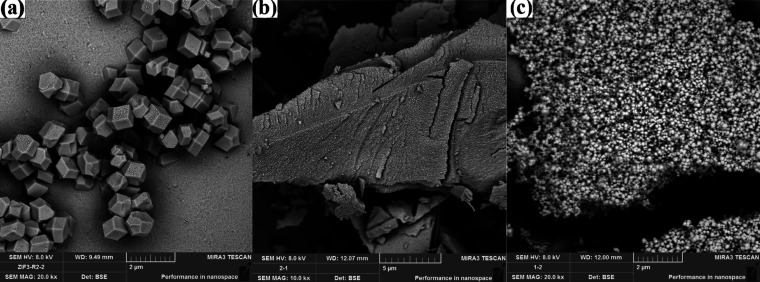
SEM images of ZIF-8/2 (a), ZIF-8/3 (b), and ZIF-8/4 (c).

Figures 1 and 2 show SEM images of ZIF-8 materials synthesized by using various drying methods and solvents. The polyhedral structure of ZIF-8 was obtained using the solvent methanol (ZIF-8/1 and ZIF-8/2). When the ZIF-8 was dried using a freeze-dryer and an oven-dryer, it was found that the drying conditions had no effect on the polyhedral structure but only on the particle size distribution (Figure 1b). For this purpose, the average particle size distribution of the polyhedral particles was determined and is shown in Figure S1. The particle size distribution of the ZIF-8/1 sample ranges from 800 to 1250 nm and is consistent with the results of Hsiao et al.^20^ The average particle size of ZIF-8 was 993.45 nm when it was dried in an oven. In addition, agglomeration of some ZIF-8/1 particles occurred, as shown in the image STEM, confirming the higher particle size of the sample. When DMF was used for the synthesis of ZIF-8, a powdery structure formed instead of the polyhedral structure of ZIF-8 (Figure 2b). SEM images in Figure 2c show that ZIF-8 forms small spherical nanoparticles with distilled water as the solvent (ZIF-8/4). However, agglomeration of the spherical particles was observed. This can be explained by the high interfacial tension between water and reactants, which provides a higher surface barrier for crystallization and delays nucleation.^4^ Therefore, the sample could not grow in water to form a polyhedral ZIF-8 structure.

The solvent used in the synthesis of ZIF-8 dissolves the zinc salt and HMIM and separates H^+^ from HMIM to form MIM. Thus, the Zn^2+^ cations combine with MIM^–^ to form ZIF-8. Moreover, the solvent was found to play an important role in stabilizing the pores during the crystallization of ZIF-8.^21^ From the literature and experimental results, methanol is a suitable solvent for the formation of polyhedral ZIF-8 crystals because methanol molecules are located near the hydrogen atoms of the MIM groups and can split hydrogen from MIM.^22,23^

**Figure 3:**
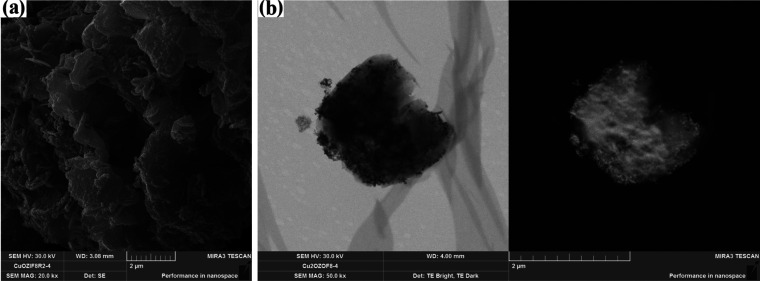
SEM (a) and STEM (b) images of Cu/ZIF-8.

**Figure 4:**
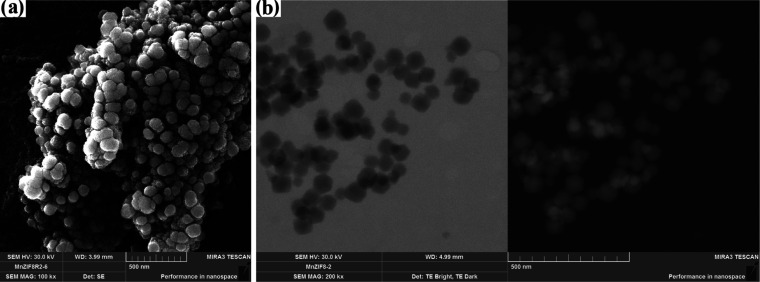
SEM (a) and STEM (b) images of Mn/ZIF-8.

According to the preparation method of ZIF-8, copper and manganese oxides and their combinations were added to the solution during the preparation of ZIF-8. The total content of metal oxides in the metal/ZIF-8 was set at 5 (mole), % since a higher metal oxide content was reported to cause deformation of the polyhedral structure.^3^ Therefore, ZIF-8 samples containing 5% Cu_2_O were prepared and characterized. The SEM and STEM images of the Cu/ZIF-8 sample in Figure 3 show the presence of intermittent polyhedral structures, in which a multilayer structure is formed. The average particle size of Cu/ZIF-8 was determined to be 33.65 nm (Figure S2) based on the STEM images. The introduction of Cu_2_O into the structure significantly decreased the particle size of the material. When ZIF-8 was doped with manganese oxide, a polyhedral structure with a smoother surface was formed, as shown in Figure 4. This structure could allow electron transport and ion penetration. A similar structure was reported for Mn/ZIF-67 sample.^19^ The average particle size of Mn/ZIF-8 was calculated to be 73.47 nm (Figure S3). A decrease in particle size can be explained by the altered coordination between Zn^2+^ and the electron-deficient pyridine nitrogen of imidazole^23,24^ in the presence of Mn^4+^. Indeed, in addition to Zn(HMIM)^2+^ complexes, Mn(HMIM)^4+^ complexes can also be formed, which can change the particle size, crystallite size, and porosity of the material. STEM images of Cu–Mn/ZIF-8 R1 and Cu–Mn/ZIF-8 R2 obtained by changing the Cu_2_O and MnO_2_ content are shown in Figures S5 and S6. It can be seen that the polyhedral structure was preserved by introducing the two metal oxides. The average particle size of the samples, Cu–Mn/ZIF-8 (R1) (2.5% Cu_2_O and 2.5% MnO_2_) and Cu–Mn/ZIF-8 (R2) (1% Cu_2_O and 4% MnO_2_), was determined to be 42.45 and 32.46 nm, respectively. With increasing MnO_2_ content in the sample, the particle size decreases.

**Figure 5:**
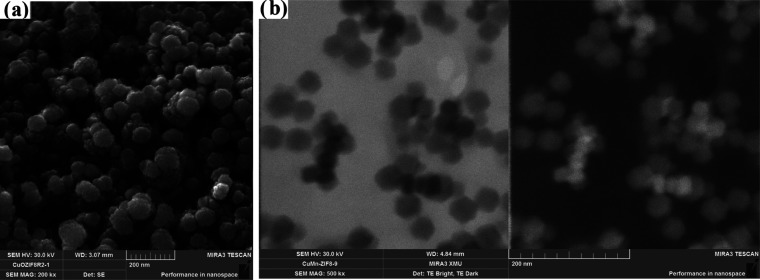
SEM (a) and STEM (b) images of Cu–Mn/ZIF-8.

To understand the effects of Cu_2_O on the structure, ZIF-8 was doped with a mixture of 4% Cu_2_O and 1% MnO_2_. SEM and STEM images of the sample in Figure 5 show that the polyhedral structure of Cu–Mn/ZIF-8 was not changed. The average particle size (43.32 nm) in Figure S4 is slightly increased compared to Cu–Mn/ZIF-8 (R2). Despite the successful integration of MnO_2_ and Cu_2_O mixtures into the ZIF-8 structure without destroying its polyhedral structure, the introduction of Cu_2_O alone changed the morphology of the composite material.

**Figure 6:**
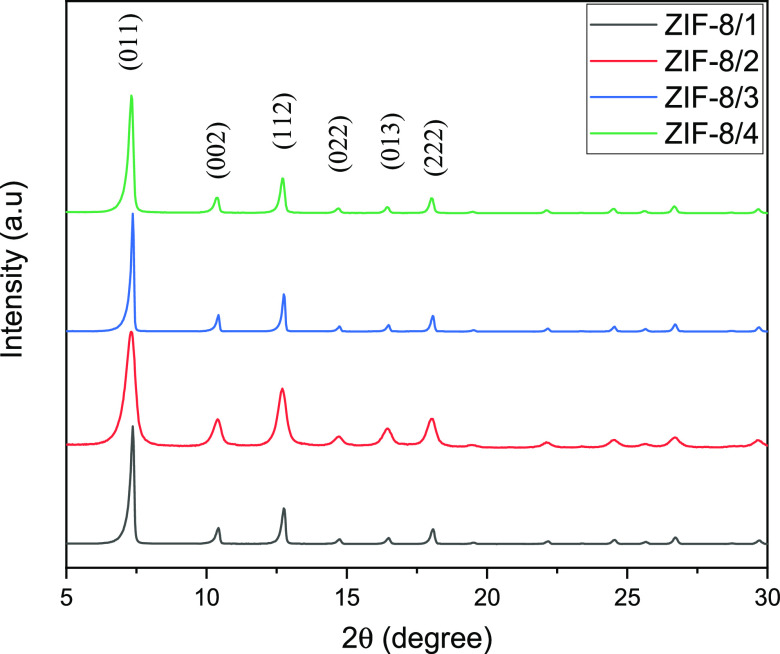
XRD graph of the ZIF-8 samples.

Figure 6 shows the XRD patterns for the synthesized ZIF-8 samples. The diffraction peaks at 2θ = 7.32, 12.76, 14.70, and 17.93° correspond to the crystal planes of points (011), (112), (022), and (222), respectively. They confirm the formation of the crystal structure of ZIF-8.^25^ The crystallite sizes of all synthesized samples are listed in Table S2. It was found that the crystallite sizes of the samples synthesized with methanol were higher than those of water and DMF. These values are consistent with the sharpness of the XRD peaks. Namely, the peaks of ZIF-8 prepared with DMF and distilled water are sharper than those of ZIF-8 synthesized with methanol. As previously noted,^26^ the kind of solvent has a considerable impact on the crystallinity of ZIF-8.

When copper oxide and manganese oxide were doped into the ZIF-8 structure, small Cu_2_O (PDF: 77-0199) and MnO_2_ (PDF: 72-1982) peaks appeared in the XRD pattern (Figure S7). The peaks indicative of ZIF-8 were also detected by metal loading, suggesting that the crystal structure was preserved.^27,28^ When the ZIF-8 structure was loaded with equal amounts of MnO_2_ and Cu_2_O, metal oxide and ZIF-8 structures were simultaneously observed in the XRD pattern. Similar structures are found for Cu/ZIF-8, Cu–Mn/ZIF-8, Cu–Mn/ZIF-8 (R1), Cu–Mn/ZIF-8 (R2), and Cu–Mn/ZIF-8 (R3). However, the addition of metal oxides to ZIF-8 resulted in a decrease in the crystallite sizes of the peaks (Table S2). For example, the addition of manganese oxide decreased the crystallite size of the material from 38.93 to 29.39 nm. A similar effect was observed for the particle size of the materials when MnO_2_ was added to ZIF-8 (see Figures S1 and S3).

**Figure 7:**
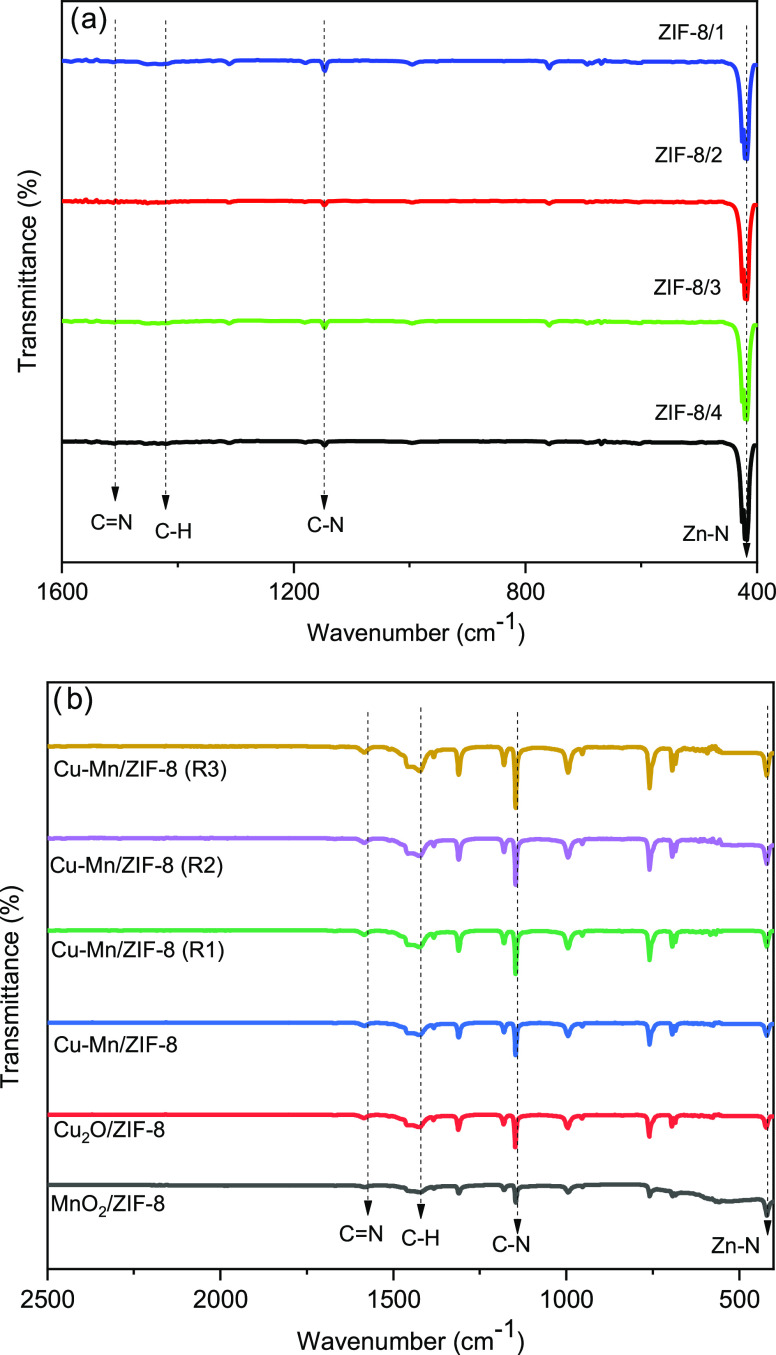
FTIR graphs of (a) ZIF-8 and (b) metal/ZIF-8.

Figure 7a shows the FTIR spectra of the ZIF-8 samples. There are ZIF-8 peaks associated with the stretching of the imidazole ring between 1500 and 1350 cm^–1^, the C=N stretching at 1590 cm^–1^, and in-plane imidazole bending (C–N) between 1350 and 900 cm^–1^. The peak at 421 cm^–1^ belongs to the vibrational strain of Zn–N, which acts as a link between zinc (Zn) and nitrogen (N).^17^ The FTIR results show that the peaks associated with the 2-MIM-NH bond at 1844 cm^–1^ cannot be detected. This can be explained by the conversion of these groups to ZIF-8, which is also reported by Hu et al.^3^ The FTIR results of the ZIF-8 samples loaded with different metals are shown in Figure 7b. After loading with the metal oxides, the functional groups of ZIF-8 changed insignificantly, and the same peaks were obtained in ZIF-8. Metal oxide peaks cannot be detected because the metal particles were embedded in the ZIF-8 crystal structure.^3^

**Figure 8:**
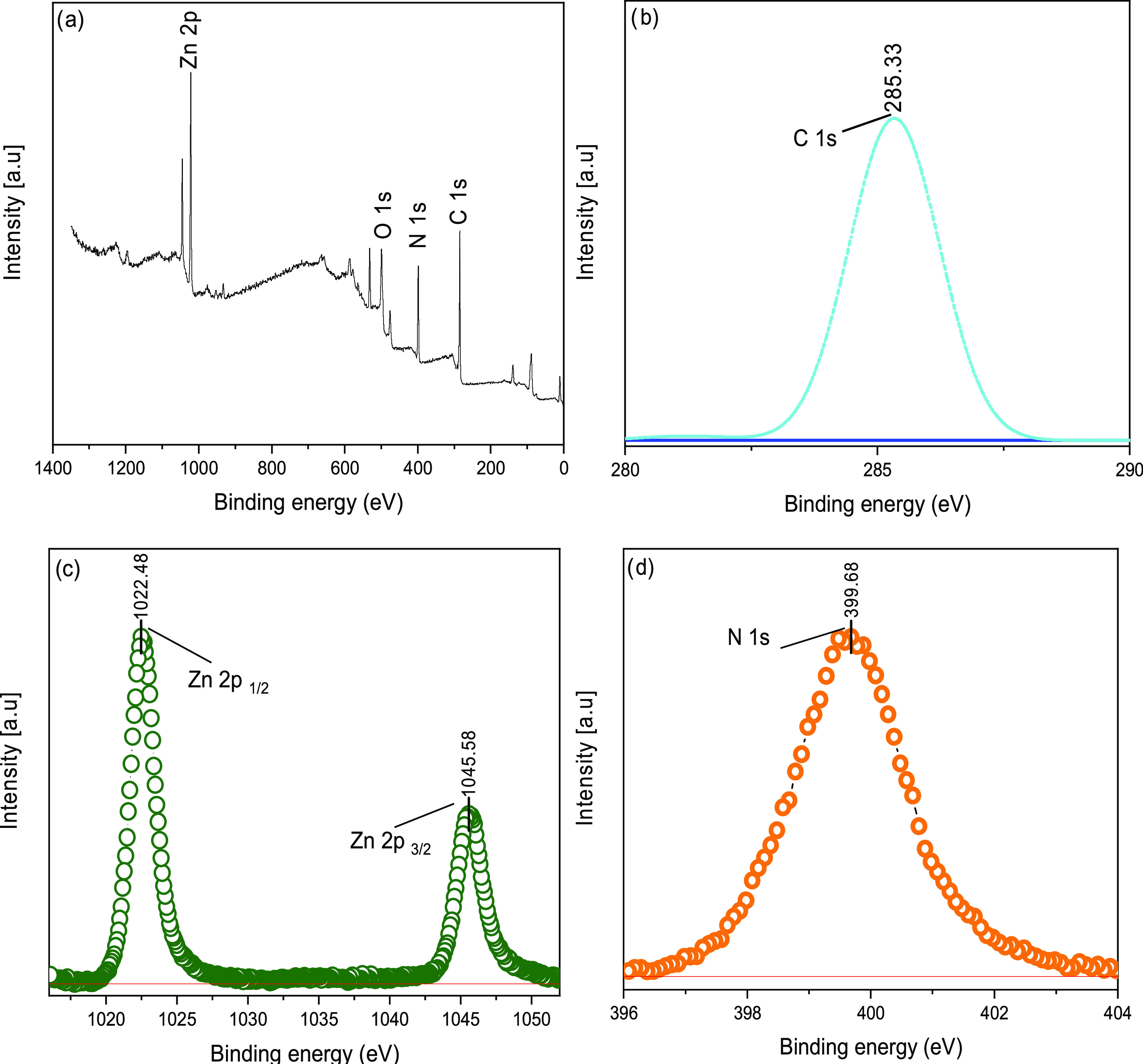
XPS survey (a), C 1s (b), Zn 2p (c), and N 1s (d) of ZIF-8.

Figure 8a shows the XPS survey spectra of the ZIF-8 sample. The bands corresponding to Zn 2p, O 1s, C 1s, and N 1s confirm the microstructure of ZIF-8. The band at 285.33 eV in the C 1s spectra (Figure 8b) of ZIF-8/1 is assigned to the C–O bond.^29^ In the Zn 2p spectra of ZIF-8/1 (Figure 8c), the Zn 2p_1/2_ and Zn 2p_3/2_ binding energies were determined at 1022.48 and 1045.58 eV, respectively. In a similar literature study, these peaks were interpreted as indicating successful bond formation between Zn^2+^ and organic ligands in the sample.^30^ This peak caused by C–N=C and C=N–C bonds supports the formation of ZIF-8. The N 1s spectra in Figure 8d show that a peak at 399.68 eV is associated with the N–Zn–N bond and indicates the presence of chemical ligands in ZIF-8.

**Figure 9:**
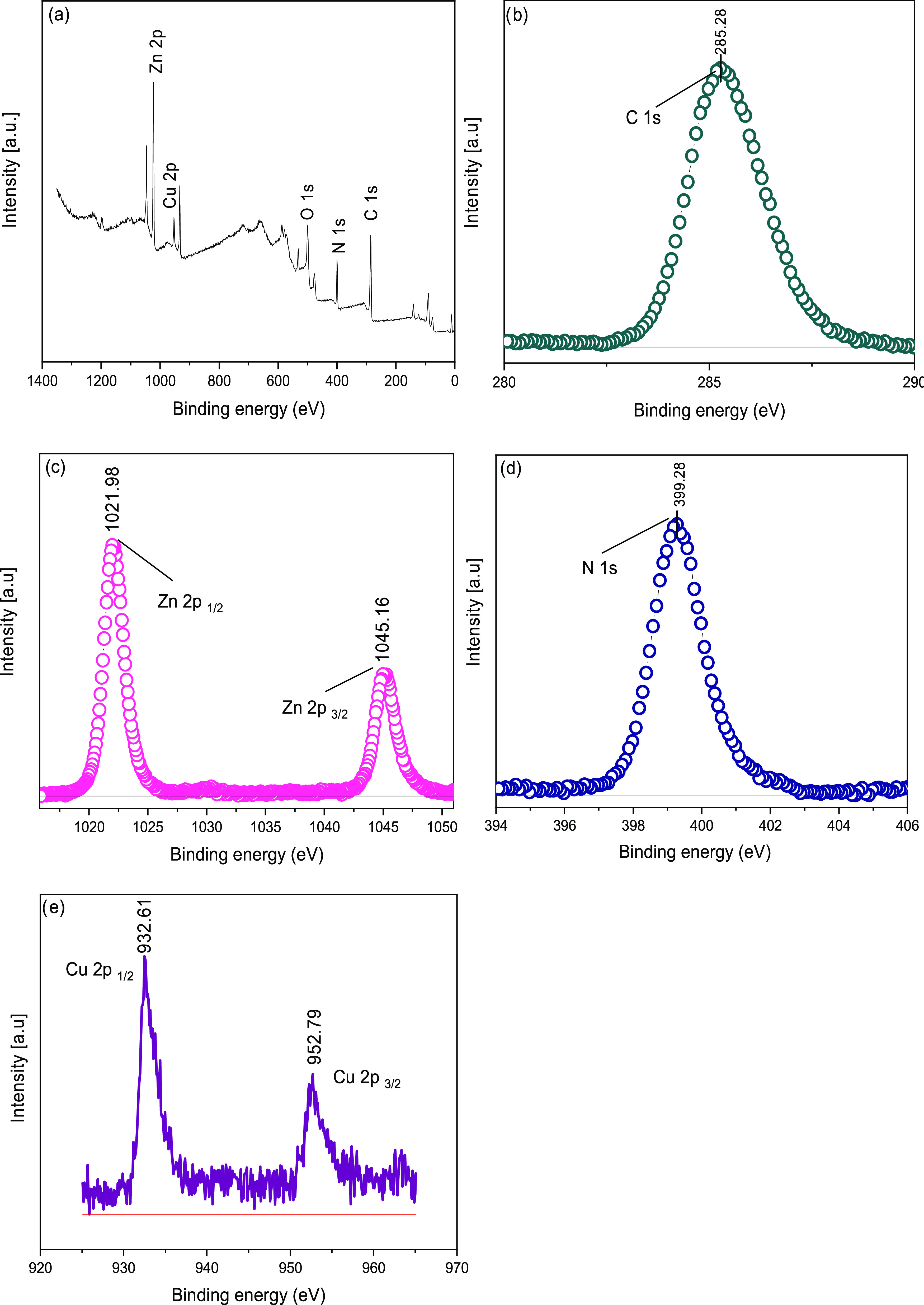
XPS survey (a), C 1s (b), Zn 2p (c), N 1s (d), and Cu 2p (e) of Cu-ZIF-8.

Figure 9a shows the XPS plot of the Cu/ZIF-8 sample. The peak at 285.28 eV is related to the C–C bond.^31^ Comparison of the XPS results of Cu/ZIF-8 with those of ZIF-8 confirms the addition of copper. The peak maximum of C 1s (Figure 9b) changed only slightly with the addition of Cu_2_O to ZIF-8. Figure 9c shows the Zn 2p curve of Cu/ZIF-8, where two peaks were detected at 1021.98 eV for Zn 2p_1/2_ and 1045.16 eV for Zn 2p_3/2_ (Table S3). The N–C bond of the imidazole ring was detected at 399.28 eV in the N 1s diagram (Figure 9d). Compared to the N 1s diagram of ZIF-8, the peaks are slightly shifted downward due to the interaction of the N–C bond with copper. Compared to the Zn 2p of ZIF-8, the maximum of the peaks is shifted to higher values, which is due to the interaction of Cu with the N–Zn–N bond. Two narrow and symmetrical peaks at 932.61 and 952.79 eV of Cu 2p are assigned to Cu(I) (Figure 9e),^32^ confirming the presence of Cu_2_O in the ZIF-8 structure as determined from XRD.

**Figure 10:**
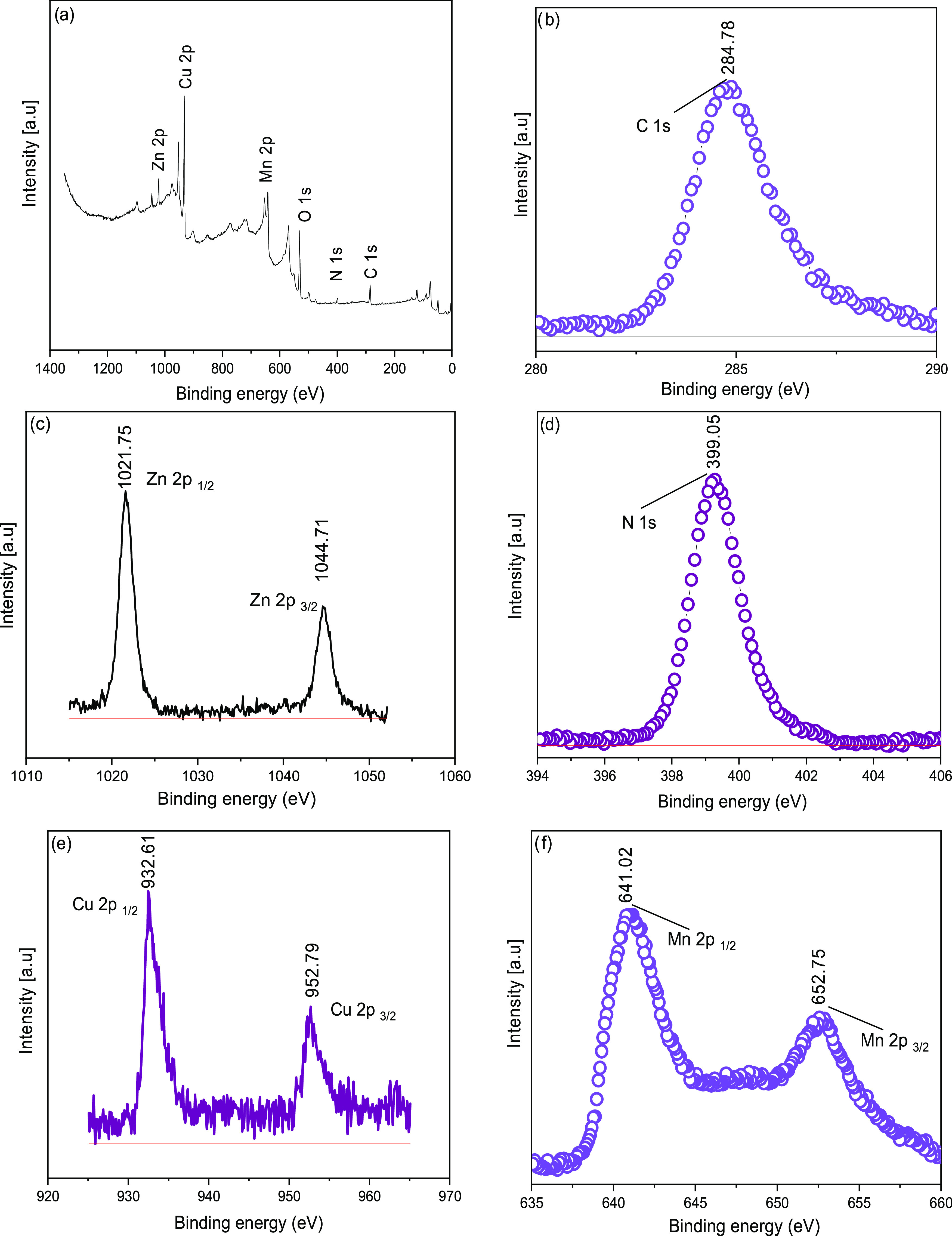
XPS survey (a), C 1s (b), Zn 2p (c), N 1s (d), Cu 2p (e), and Mn 2p (f) of Cu–Mn/ZIF-8.

The XPS survey spectra of Cu–Mn/ZIF-8 are shown in Figure 10a. The sample consists of C, N, Zn, Cu, Mn, and O. The C 1s spectra show that the peak at 284.78 eV is related to the C–C bond (Figure 10b). For Zn 2p, Zn 2p_3/2_ at 1044.71 eV and Zn 2p_1/2_ at 1021.75 eV (Table S3) are detected in Figure 10c. In the N 1s XPS spectra, the peak at 399.05 eV refers to the N–Zn–N bond (Figure 10d). The Cu 2p peaks in Figure 10e show the presence of Cu 2p_3/2_ at 952.79 eV and Cu 2p_1/2_ at 932.61 eV. Two peaks for Mn 2p in Figure 10f were observed at 652.75 eV for Mn 2p_3/2_ and 641.02 eV for Mn 2p_1/2_.^19^ The binding energy value between the Mn 2p_3/2_ and Mn 2p_1/2_ peaks is 11.7 eV, indicating the presence of MnO_2_.^33^ Moreover, the incorporation of Cu and Mn into the ZIF-8 structure leads to a shift in the ZIF-8 peaks, confirming the existence of both metal oxides in the Cu–Mn/ZIF-8 structure. The insertion of Cu and Mn into the ZIF-8 structure leads to a shift in the ZIF-8 peaks, confirming the existence of both metal oxides in the Cu–Mn/ZIF-8 structure. In addition, when the XPS analysis of the Cu_2_O/ZIF-8 sample is compared, it is seen that the binding energies of the Cu 2p element do not change. These peaks indicate that Cu^+^ is present in the Cu–Mn/ZIF-8 mixed oxide sample and the spinel structure is formed due to the synergistic effect independent of the Cu/Mn mole ratios. With the presence of spinel structure, Cu^2+^ and Mn^3+^ are also present in the structure (Cu^2+^ + Mn^3+^ ↔ Cu^+^ + Mn^4+^) as well as Cu^+^ and Mn^4+^ ions.^34^

XPS plot for the Mn/ZIF-8 sample is illustrated in Figure S8a. The peaks of C 1s (Figure S8b), N 1s (Figure S8c), Zn 2p_1/2_, and Zn 2p_3/2_ (Figure S8d) resulting from the bonds between organic ligands and zinc nitrate hexahydrate were determined to be 285.08, 399.08, 1022.08, and 1045.07 eV, respectively. Since the amount of manganese in ZIF-8 is low, no peak could be detected. The peak maxima of carbon, nitrogen, and zinc changed insignificantly. However, the peaks of copper and manganese could not be detected because of a small amount in the sample.

XPS survey of the Cu–Mn/ZIF-8 (R2) sample is shown in Figure S9a. The peaks of C 1s (Figure S9b), N 1s (Figure S9c), Zn 2p_1/2_, and Zn 2p_3/2_ (Figure S9d) were determined to be 285.08, 399.18, 1022.18, and 1045.28 eV, respectively, showing that the structure of ZIF-8 is preserved by the addition of copper and manganese oxides.

**Figure 11:**
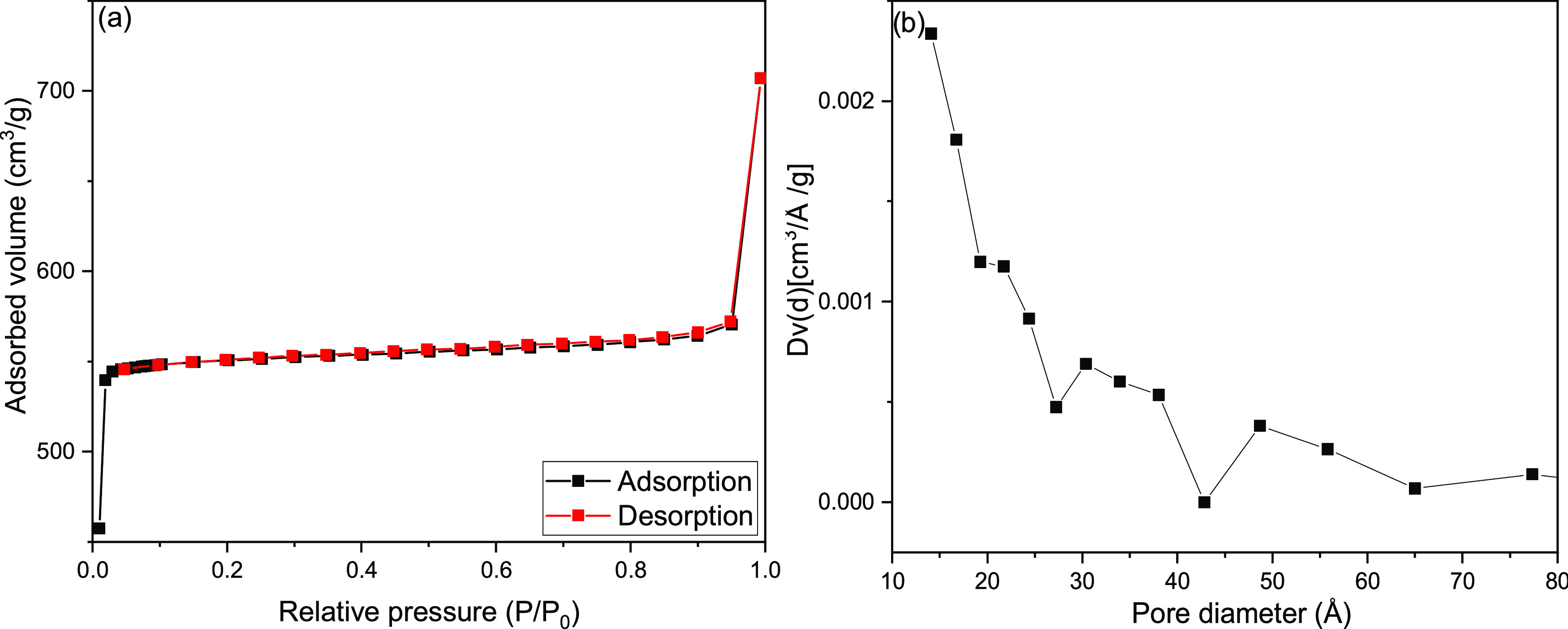
Isotherm (a) and pore distribution (b) of ZIF-8.

The adsorption–desorption isotherm with N_2_ and the pore size distribution of ZIF-8 are shown in Figure 11. ZIF-8 showed a type I isotherm, which is a monolayer adsorption according to the IUPAC classification.^35^ The pore size distribution of ZIF-8 in Figure 11b indicates the formation of mesopores by the presence of peaks at 500 Å > *D*_p_ > 20 Å along with micropores at *D*_p_ < 20 Å. The surface properties in Table 1 show that the surface area, micropore volume, and average pore diameter of ZIF-8 are 2088 m^2^/g, 0.84 cm^3^/g, and 21.0 Å, respectively. The higher surface area and porosity values of ZIF-8 are related to the large kinetic energies and fast diffusion rates of Zn^2+^ and MIM ions.^4^

**Table 1:** Surface Area and Pore Characteristics of ZIF-8 and Metal/ZIF-8 Samples

sample	SA (m^2^/g)^a^	*V*_T_ (cm^3^/g)^b^	*D* (Å)^c^	*V*_micro_ (cm^3^/g)^d^
ZIF-8	2088.0	0.2	21.0	0.84
Cu/ZIF-8	462.3	0.25	33.78	0.16
Mn/ZIF-8	1408.0	0.72	34.82	0.52
Cu–Mn/ZIF-8	2084.0	1.14	35.0	0.77
Cu–Mn/ZIF-8 R1	1464.0	1.13	51.0	0.82
Cu–Mn/ZIF-8 R2	1996.0	1.11	36.78	0.79
Cu–Mn/ZIF-8 R3	1870.0	0.78	30.76	0.72

a Surface area determined by multipoint BET.

b Total pore volume determined at *P*/*P*^o^ = 0.99.

c Average pore diameter determined by *t*-plot.

d Micropore volume determined by *t*-plot.

The N_2_ adsorption–desorption isotherm for the Cu/ZIF-8 sample is shown in Figure S10a. The isotherm is consistent with a type IV isotherm. The hysteresis between the adsorption and desorption curves in the range of 0.6–1.0 *P*/*P*^o^ is related to the formation of mesopores. The pore size distribution of ZIF-8 shows the presence of micropores by the detection of peaks at *D*_p_ < 20 Å in Figure S10b. The Cu/ZIF-8 sample had a surface area of 462.3 m^2^/g and an average pore size of 33.78 Å (Table 1). It was found that the addition of Cu_2_O to ZIF-8 decreased the surface area and pore volume due to the accumulation of Cu_2_O particles in the pores (Figure S7b) and the change of the structure from a polyhedral structure to a layered structure (Figure 3).

Based on the isotherm and pore size distribution (Figure S11a,b), the Mn/ZIF-8 sample shows isotherms of type IV, consisting of micro- and mesopores. The calculated surface properties of the material are a surface area of 1408.0 m^2^/g, a pore volume of 0.72 cm^3^/g, and a pore diameter of 34.82 Å. The comparison of Mn/ZIF-8 with ZIF-8 showed a decrease in surface area and pore diameter. The decrease in the surface area could be due to the accumulation of manganese oxide particles in the pores of ZIF-8, as shown in Figure 4a.

**Figure 12:**
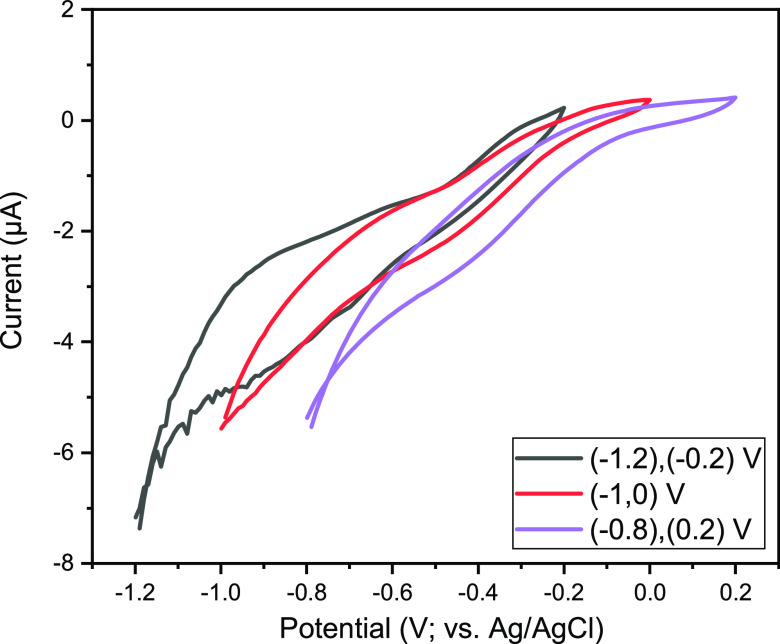
CV curve of Cu–Mn/ZIF-8 at different potential ranges.

The isotherm and pore size distribution of the Cu–Mn/ZIF-8 sample (the molar ratio of Cu to Mn is 4:1) are shown in Figure S12. The isotherm of Cu–Mn/ZIF-8 shown in Figure S12a corresponds to type V and shows the formation of mesopores and mesopores (Figure 12b). The total pore volume, average pore diameter, and surface area of Cu–Mn/ZIF-8 were calculated to be 1.14 cm^3^/g, 35.0 Å, and 2084 m^2^/g, respectively. By increasing the Cu content and decreasing the Mn content in ZIF-8, the Cu–Mn/ZIF-8 (R1) (the molar content of Cu and Mn is 2.5:2.5) (Figure S13), the surface area decreased to 1464.0 m^2^/g and the average pore volume increased to 51.0 Å. When the MnO_2_ content was further increased and the Cu_2_O content was decreased, Cu–Mn/ZIF-8 R2 (Figure S14), which was prepared by adding 4% MnO_2_ and 1% Cu_2_O, showed an opposite trend to Cu–Mn/ZIF-8 (R1) by increasing the surface area (1996.0 m^2^/g) and decreasing the average pore diameter (36.78 Å). In addition, Cu–Mn/ZIF-8 R3 with 2% Cu_2_O and 3% MnO_2_ was synthesized and characterized (Figure S15). The surface area, total pore volume, micropore volume, and crystallite size were determined to be 1870 m^2^/g, 0.78 cm^3^/g, 0.72 cm^3^/g, and 22.5 nm, respectively.

The study of the influence of Cu and Mn ratio showed that the increasing concentration of Cu_2_O leads to a significant reduction of the surface area and pores because the polyhedral structure tends to a layered structure and Cu_2_O particles accumulate in the pores. The addition of MnO_2_, on the other hand, limits the reduction of the surface area considerably since the crystal size decreases together with the diameter of the polyhedral particles.

### 3.2 Electrochemical Measurement

The CV measurements of the synthesized samples were performed at different potential windows ranging from −1.2 to −0.2 V, −1.0 to 0.0 V, and −0.8 to 0.2 V at a constant scan rate of 5 mV/s (Figure S16). The areas of the CV curves are exactly proportional to the specific capacitance of the electrode, as shown in eq 1. As can be seen for the Cu–Mn/ZIF-8 sample, a slightly higher value for the specific capacitance was obtained, which can be attributed to the higher pseudocapacitive performance of Cu–Mn deposited on ZIF-8. The electrodes exhibited a faradaic transition and a reversible redox response when scan rates were increased. As a result, the current values increased.

Among the potential windows, the potential range from −1.2 to −0.2 provides the best value for the specific capacitance for all samples. The specific capacitance values for ZIF-8, Cu/ZIF-8, Mn/ZIF-8, and Cu–Mn/ZIF-8 samples in these potential ranges were determined to be 30.13, 33.74, 52.32, and 56.37 (F/g), respectively. Cu–Mn/ZIF-8 (Figure 12) has a higher specific capacitance. This could be related to the increase in electrochemical performance due to the synergistic effect of copper and manganese ions.^36^

**Figure 13:**
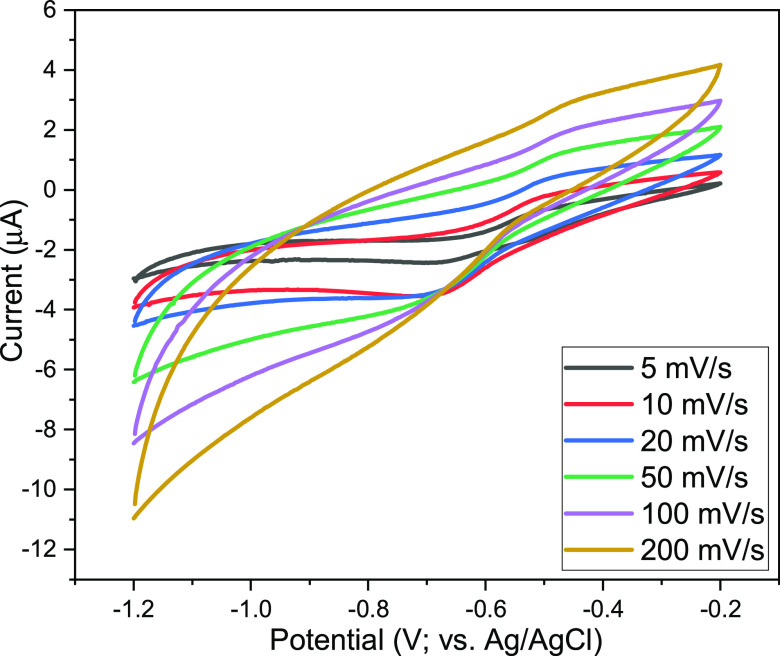
CV curve of Cu–Mn/ZIF-8 at different scan rates.

Measurements for all samples were performed at different sampling rates (5, 10, 20, 50, 100, and 200 mV/s) between (−1.2) and (−0.2), as shown in Figure 13. CV curves of all electrodes show redox peaks due to reversible redox reactions of the active material. During the charging and discharging process, the redox mechanisms of ZIF-8^37^ and Cu/ZIF-8^38^ are shown as follows
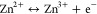
2
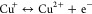
3There are two basic mechanisms for supercapacitive charge storage in MnO_2_. The first is based on the theory of adsorption/desorption of the metal cation in the electrolyte (eq 4), while the other mechanism is based on the intercalation/extraction of alkali cations into oxide particles (eq 5). In both mechanisms, the reversible redox reaction and the oxidation state of manganese alternate between III and IV.^39^
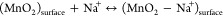
4
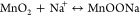
5

**Figure 14:**
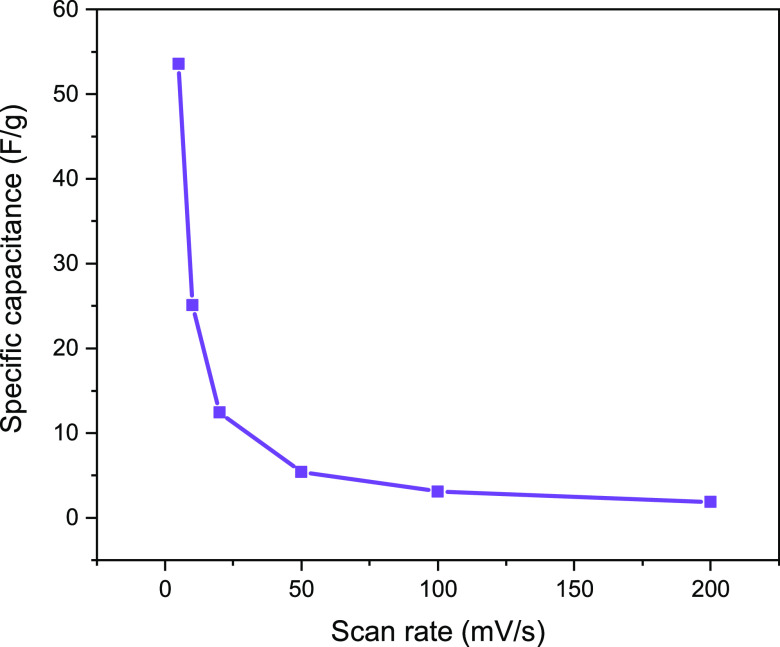
Capacitance variation with a scan rate of Cu–Mn/ZIF-8.

Figure S17 shows the specific capacitance of all samples as a function of the different scan rates. As the scan rate increased, the current response values increased, indicating good and stable behavior of the capacitor. The specific capacitance was found to decrease with an increasing scan rate (Figure 14). The diffusion limitations of the ions lead to a reduction in the ion contacts within the electrode material, which limits the interaction of the ions with the electrode material and leads to a lower specific capacitance at higher scan rates. Analysis of the individual capacitance values showed that the addition of 1% MnO_2_ to this material increased the peak current range compared to that of the Cu/ZIF-8 sample. This can also be explained by the larger surface area of Cu–Mn/ZIF-8, which allows for easier penetration of the electrolyte solution into the pores, as shown in Table 1.

The results of the electrochemical analysis showed that the specific capacitance of bare ZIF-8 was 30.13 F/g, which could be due to the low conductivity, which is one of the disadvantages of the ZIF-8 structure. While the specific capacitance changed insignificantly with the loading of copper oxide on the ZIF-8 structure, a significant improvement in the specific capacitance was observed with the loading of manganese. The electrochemical analysis results of ZIF-8 showed that the Cu–Mn/ZIF-8 with 4% Cu_2_O and 1% MnO_2_ had the highest value for specific capacitance (56.37 F/g) (Table 2). The specific capacitance decreased to 35.26 F/g when the mole fractions of copper and manganese oxide remained the same (R1). This can be explained by the increase in manganese content in the sample. Increasing the MnO_2_ content in ZIF-8 slightly improved the capacitance because ion transfer was facilitated by increasing the surface area (Table 1) and decreasing the particle size (Figure S6). In conclusion, the presence of metal oxide in the ZIF-8 structure improved the specific capacitance with its pseudocapacitance properties depending on the surface area and porosity of the material.

**Table 2:** Specific Capacitance of Metal/ZIF-8 Samples

Sample	Specific capacitance (F/g)
ZIF-8	30.13
Cu/ZIF-8	33.74
Mn/ZIF-8	52.32
Cu–Mn/ZIF-8	56.37
Cu–Mn/ZIF-8 (R1)	35.26
Cu–Mn/ZIF-8 (R2)	48.71
Cu–Mn/ZIF-8 (R3)	36.42

## 4 Conclusions

In this study, the synthesis and characterization of ZIF-8 and metal oxide/ZIF-8 were performed. SEM images of the ZIF-8 samples synthesized with different solvents showed that a polyhedral structure of ZIF-8 was obtained with methanol, while the ZIF-8 samples prepared with DMF and distilled water were obtained agglomerated particles. In addition, MnO_2_, Cu_2_O, and a mixture of both metal oxides were successfully loaded without destroying the ZIF-8 structure. The introduction of copper or manganese oxide into the ZIF-8 structure significantly decreased its surface area and pore volume depending on the type and ratio of metal oxides. The highest surface area was found to be 2084 m^2^/g for ZIF-8 with 2.5% Cu_2_O and 2.5% MnO_2_. The XPS analysis of ZIF-8 and metal/ZIF-8 samples confirmed the formation of bonds in the crystal structure of ZIF-8 and the introduction of metal oxides into the crystal structure of ZIF-8, respectively.

Electrochemical studies showed that the introduction of Cu and Mn into the structure of ZIF-8 slightly improved the capacitance of ZIF-8. The Mn/ZIF-8 sample showed a higher specific capacitance than Cu/ZIF-8. The doping of Mn and Cu at different ratios in ZIF-8 affected the electrochemical performance, and it was found that ZIF-8 with 1% MnO_2_ and 4% Cu_2_O exhibited better capacitance values due to the synergistic effect of manganese oxide with high specific capacitance and copper oxide with higher electrical conductivity. Based on the characterization results, ZIF-8 and metal/ZIF-8 materials provide opportunities for various applications such as catalysts, gas storage, membranes, adsorption, etc.

## Supporting Information Available

Table of EDS and XRF elemental analysis of ZIF-8 and metal/ZIF-8 samples; STEM images of ZIF-8, Cu_2_O/ZIF-8, MnO_2_/ZIF-8, Cu–Mn/ZIF-8, Cu–Mn/ZIF-8 (R1), and Cu–Mn/ZIF-8 (R2); XRD graph and crystallite sizes table of Cu_2_O/ZIF-8, MnO_2_/ZIF-8, Cu–Mn/ZIF-8, Cu–Mn/ZIF-8 (R1), Cu–Mn/ZIF-8 (R2), and Cu–Mn/ZIF-8 (R3); XPS survey and data table of MnO_2_/ZIF-8 and Cu–Mn/ZIF-8 (R2); and CV curves of ZIF-8, Cu_2_O/ZIF-8, MnO_2_/ZIF-8, Cu–Mn/ZIF-8, Cu–Mn/ZIF-8 (R1), Cu–Mn/ZIF-8 (R2), and Cu–Mn/ZIF-8 (R3) (PDF)
