# Supplementary material for: Preparation, Characterization, and Application of Metal Oxide-Doped Zeolitic Imidazolate Framework
Source: ACS Omega. 2023 Jul 20;8(30):27650–62. doi: 10.1021/acsomega.3c03509 (PMC10398871; doi:10.1021/acsomega.3c03509)
Supplement: Supplementary file 1 — ao3c03509_si_001.pdf [file ao3c03509_si_001.pdf]

Preparation, characterization and application of metal oxide doped zeolitic imidazolate framework

Fulya Kümbetlioğlu[a], Kürşad Oğuz Oskay[b], Zafer Çıplak[a] and Ayten Ateş[a*]

[a]Sivas Cumhuriyet University, Faculty of Engineering, Department of Chemical Engineering, Sivas, Turkey

[b]Sivas Cumhuriyet University, Faculty of Engineering, Department of Metallurgical and Materials Engineering, Sivas, Turkey

*Corresponding author current address: Sivas Cumhuriyet University, Engineering Faculty, Department of Chemical Engineering, 58140 Sivas, Turkey. E-mail: ates@cumhuriyet.edu.tr; Tel: +90 2191010/2248; Fax:+903462191165

Table of Contents Page

Figure S 1.STEM image of ZIF-8

Figure S 2. STEM image of Cu/ZIF-8

Figure S 3.STEM image of Mn/ZIF-8

Figure S 4. STEM image of Cu-Mn/ZIF-8

Figure S 5. STEM image of Cu-Mn/ZIF-8 (R1)

Figure S 6. STEM image of Cu-Mn/ZIF-8 (R2)

Figure S 7. XRD graph of a) Cu_2O/ZIF-8 b) MnO_2/ZIF-8 c) Cu-Mn/ZIF-8 d) Cu-Mn/ZIF-8 (R1) e) Cu-Mn/ZIF-8 (R2) f) Cu-Mn/ZIF-8 (R3) g) all Cu-Mn/ZIF-8 samples

Figure S 8.XPS survey (a), C 1s (b), N 1s (c), Zn 2p (d) of Mn/ZIF-8

Figure S 9.XPS survey (a), C 1s (b), N 1s (c), Zn 2p (d) of Cu-Mn/ZIF-8 (R2)

Figure S 10**.**CV curves of a) Cu/ZIF-8 b) Mn/ZIF-8 at different potential windows (-1.2 - –0.2 V; -1.0 – 0.0 V; -0.8 – 0.2 V) at 5 mV/s

Figure S 11**.**CV curves of a) ZIF-8 b) Cu/ZIF-8 c) Mn/ZIF-8 d) Cu-Mn/ZIF-8 R1 e) Cu-Mn/ZIF-8 R2 f) Cu-Mn/ZIF-8 R3 at different scan rates (5-200 mV/s)

Table S1. EDS and XRF elemental analysis of ZIF-8 and metal/ZIF-8 samples
Table S2. Crystallite sizes of all synthesized samples
Table S3. Peak maximum determined from XPS data of samples

Table S1. EDS and XRF elemental analysis of ZIF-8 and metal/ZIF-8 samples

Sample	C	N	O	Zn	Cu	Mn
ZIF-8	41.61	22.59	1.00	34.47	-	-
Cu_2O/ZIF-8	22.85	8.95	9.35	41.40	17.46	-
MnO_2/ZIF-8	33.64	18.08	12.95	22.61	-	12.71
Cu-Mn/ZIF-8	42.30	26.70	1.16	29.39	1.14*	0.72
Cu-Mn/ZIF-8 (R1)	42.13	27.16	1.89	28.04	0.66*	1.03
Cu-Mn/ZIF-8 (R2)	42.35	27.04	2.28	28.12	0.27*	0.56
Cu-Mn/ZIF-8 (R3)	42.60	27.16	2.78	27.00	0.29	0.35*

*Composition determined by XRF

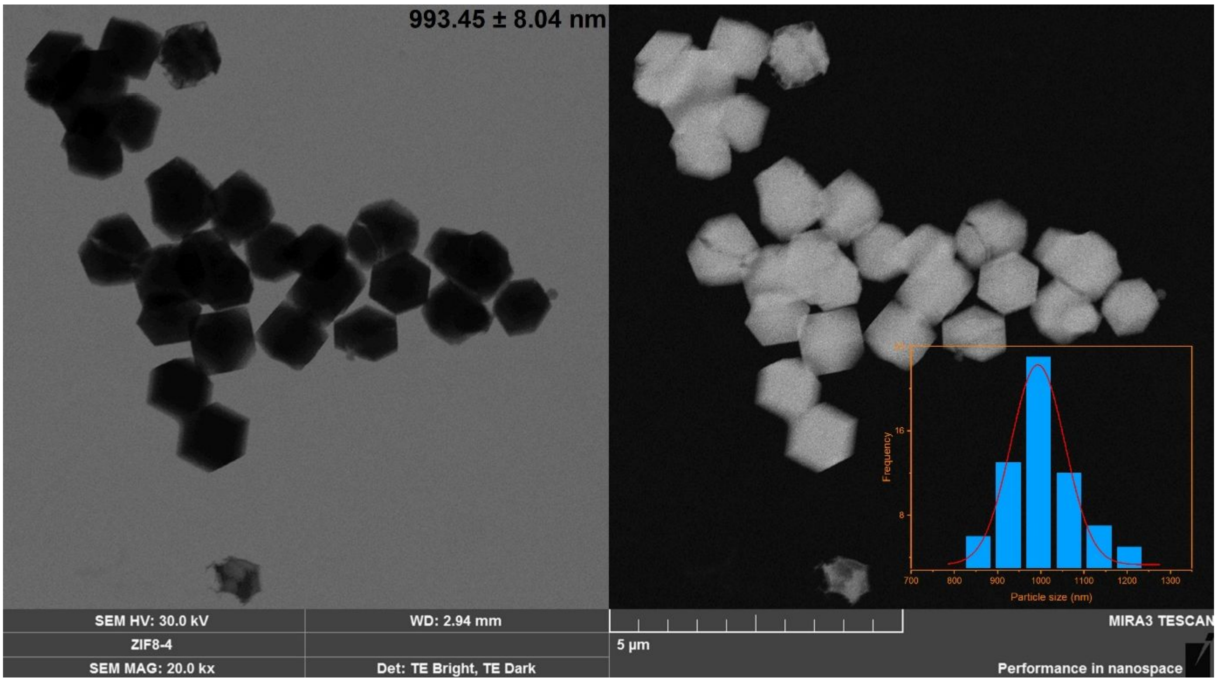

Figure S1.STEM image of ZIF-8

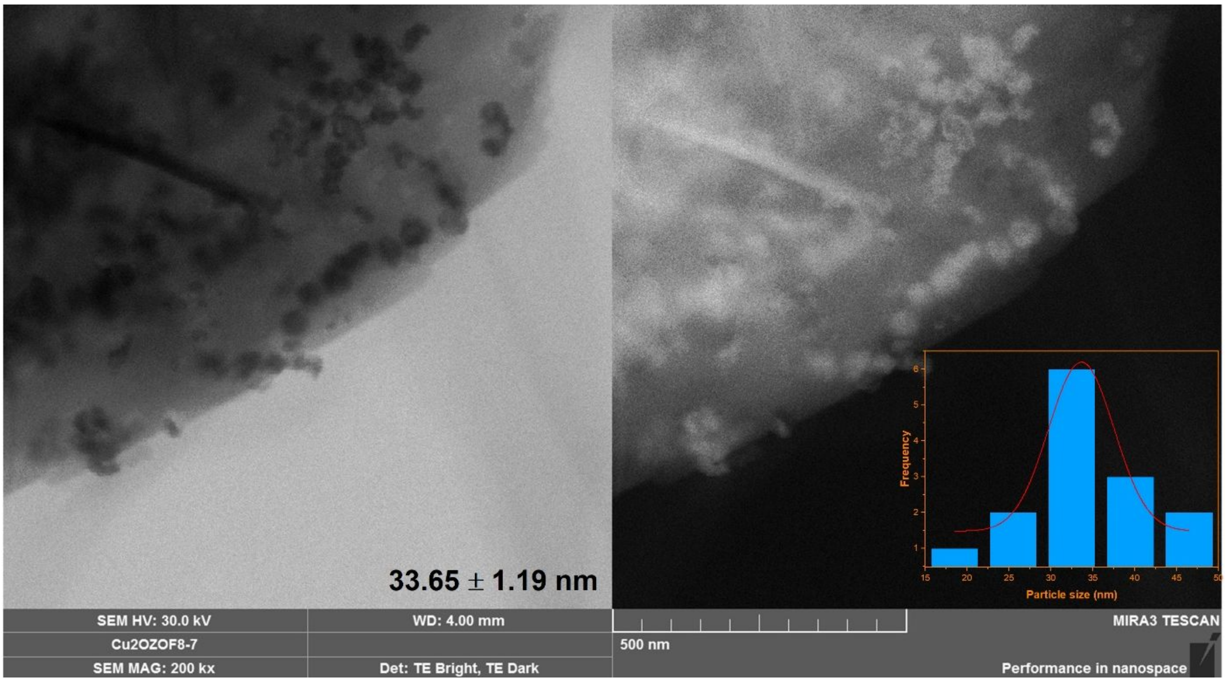

Figure S2. STEM image of Cu/ZIF-8

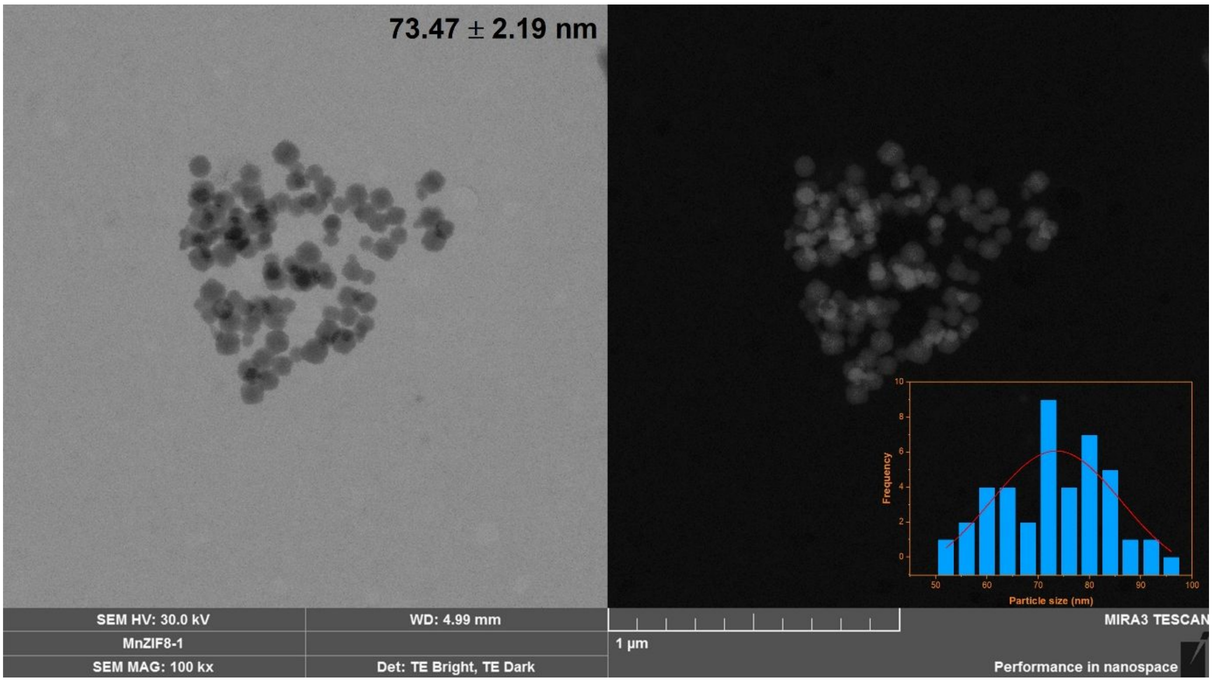

Figure S3.STEM image of Mn/ZIF-8

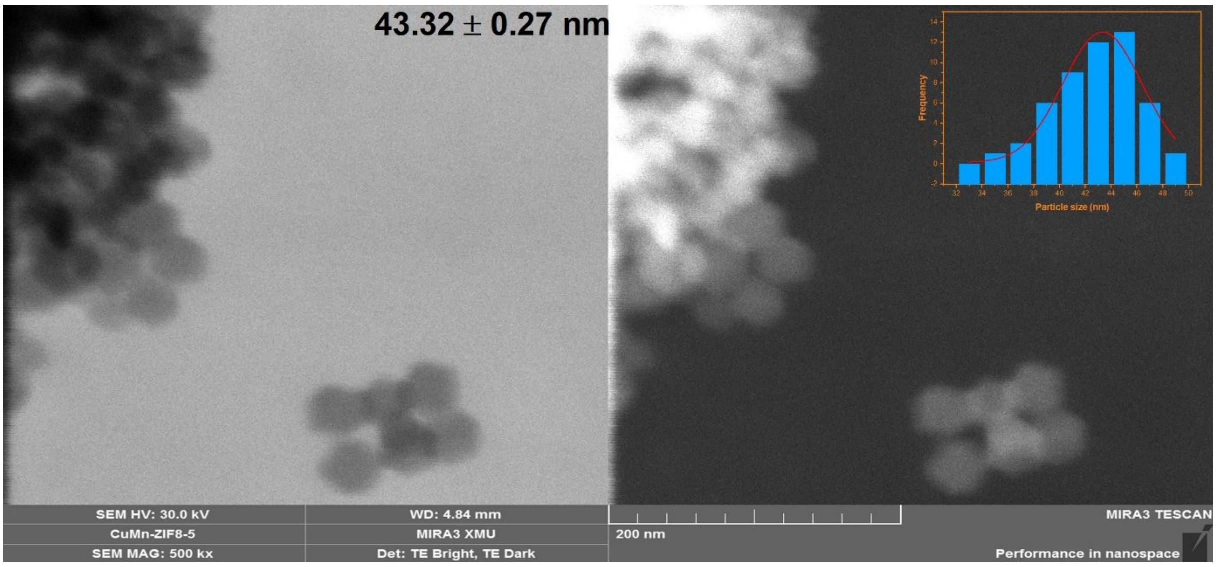

Figure S4. STEM image of Cu-Mn/ZIF-8

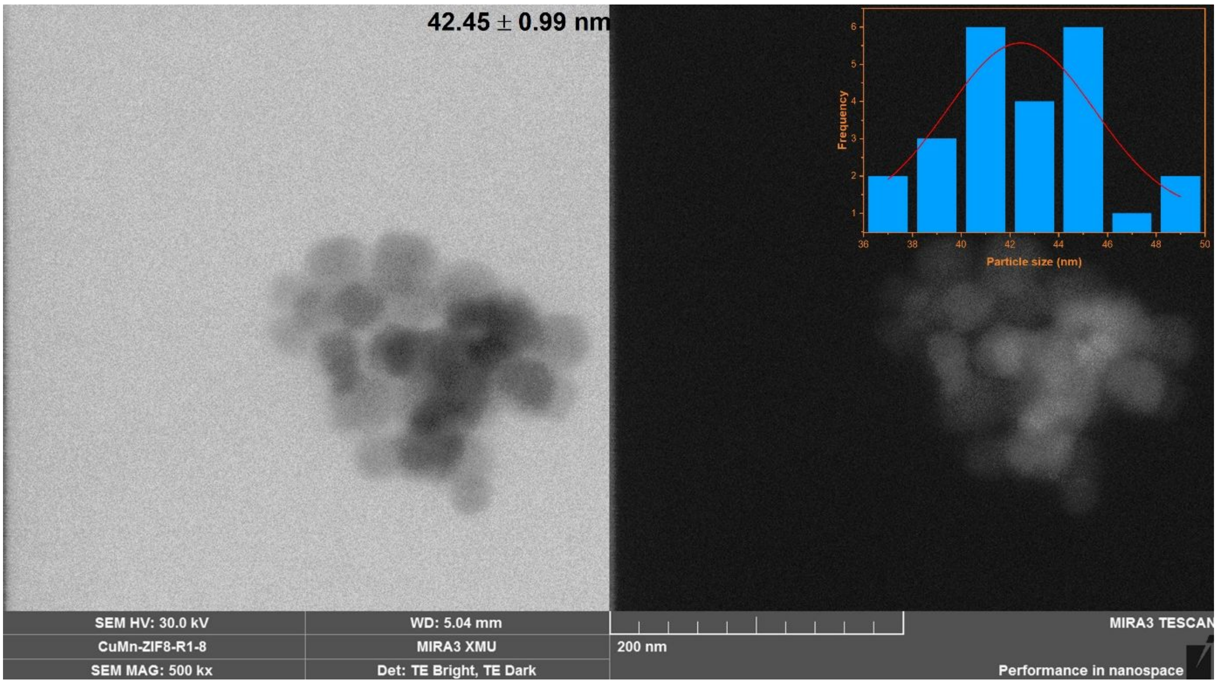

Figure S5. STEM image of Cu-Mn/ZIF-8 (R1)

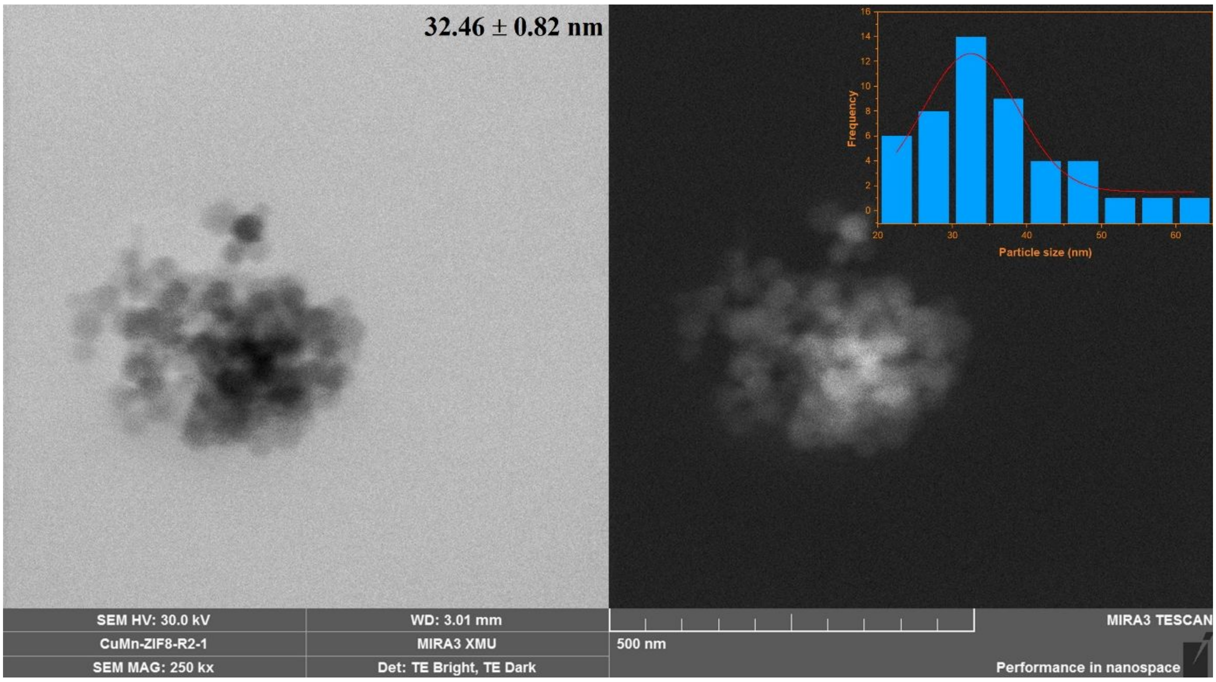

Figure S6. STEM image of Cu-Mn/ZIF-8 (R2)

Crystallite sizes of all sythesized samples were calculated from Scherrer Eq. (S1):

$$D = \frac{k\lambda}{\beta_{hkl}\cos\theta_{hkl}} \quad \text{(S1)}$$

where λ is the X-ray wavelength (0.154 nm), β is the peak width of the diffraction peak profile at half maximum height resulting from small crystallite size in radians and k is a constant related to crystallite shape, for ZIF-8 taken as 0.94.

Table S2. Crystallite sizes of all synthesized samples

**Sample**	**Crystallite size (nm)**
ZIF-8/1	43.48
ZIF-8/2	38.93
ZIF-8/3	17.33
ZIF-8/4	34.82
Cu/ZIF-8	33.08
Mn/ZIF-8	29.39
Cu-Mn/ZIF-8	21.91
Cu-Mn/ZIF-8 (R1)	22.40
Cu-Mn/ZIF-8 (R2)	22.99
Cu-Mn/ZIF-8 (R3)	22.50

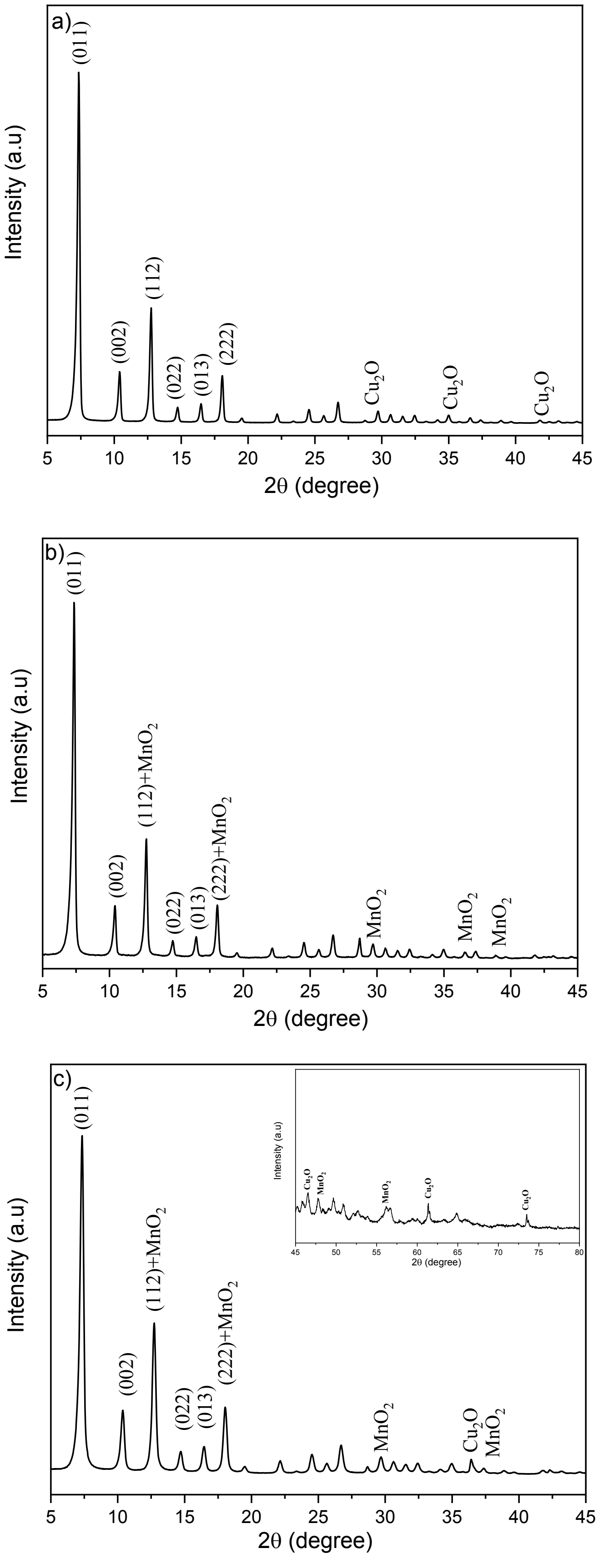
a)
(011)
(002)
(112)
(022)
(013)
(222)
Cu2O
Cu2O
Cu2O
Intensity (a.u)
2θ (degree)
b)
(011)
(002)
(112)+MnO2
(022)
(013)
(222)+MnO2
MnO2
MnO2
MnO2
Intensity (a.u)
2θ (degree)
c)
(011)
(002)
(112)+MnO2
(022)
(013)
(222)+MnO2
MnO2
Cu2O
MnO2
Intensity (a.u)
2θ (degree)

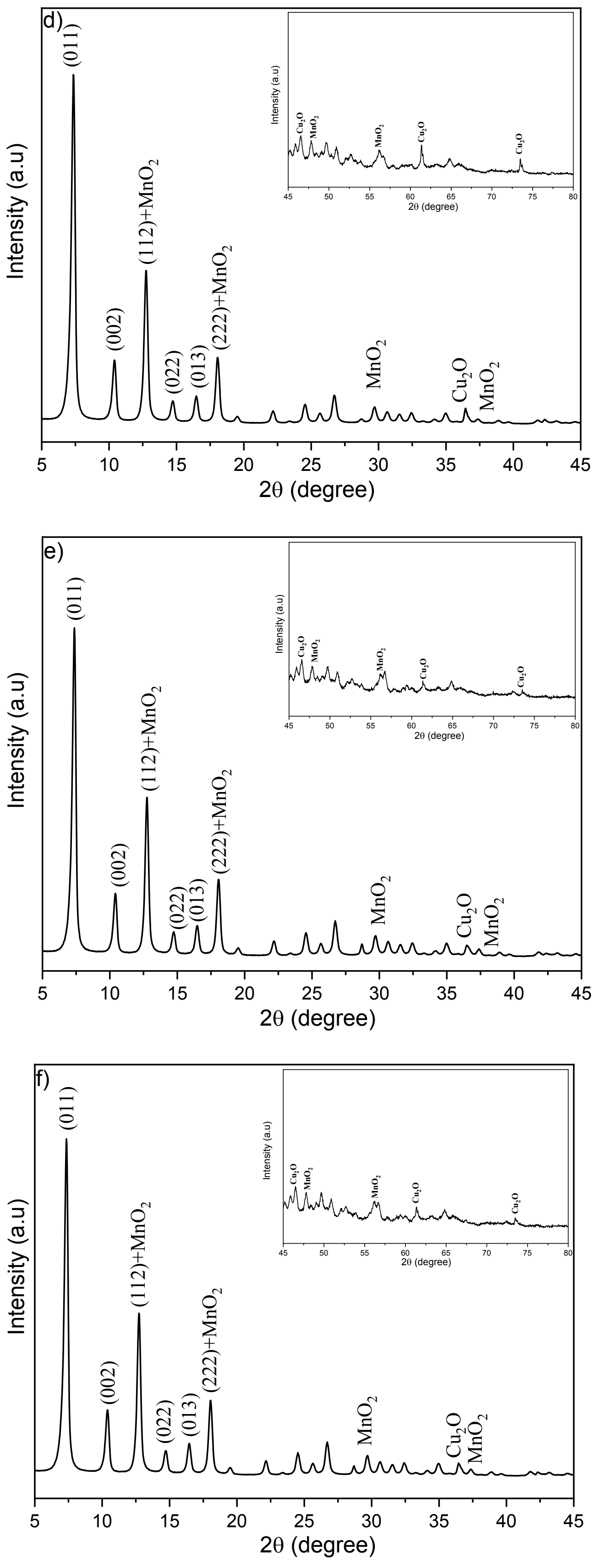
d)
(011)
(002)
(112)+MnO2
(022)
(013)
(222)+MnO2
MnO2
Cu2O
MnO2
Intensity (a.u)
2θ (degree)
e)
(011)
(002)
(112)+MnO2
(022)
(013)
(222)+MnO2
MnO2
Cu2O
MnO2
Intensity (a.u)
2θ (degree)
f)
(011)
(002)
(112)+MnO2
(022)
(013)
(222)+MnO2
MnO2
Cu2O
MnO2
Intensity (a.u)
2θ (degree)

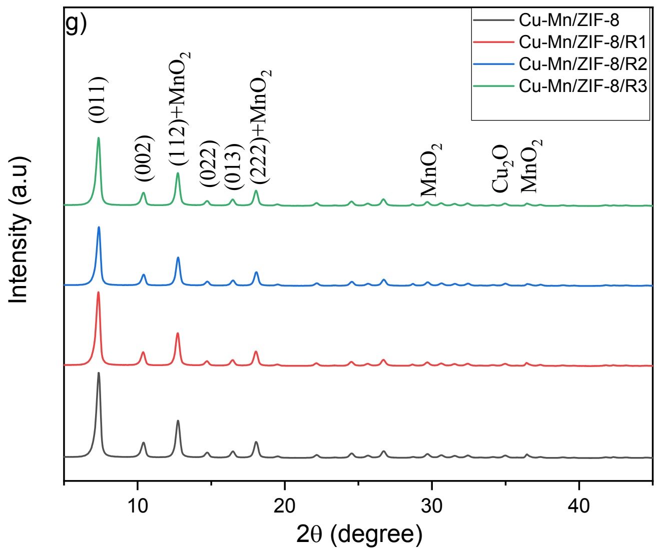

Figure S7. XRD graph of a) Cu_2O/ZIF-8 b) MnO_2/ZIF-8 c) Cu-Mn/ZIF-8 d) Cu-Mn/ZIF-8 (R1) e) Cu-Mn/ZIF-8 (R2) f) Cu-Mn/ZIF-8 (R3) g) all Cu-Mn/ZIF-8 samples

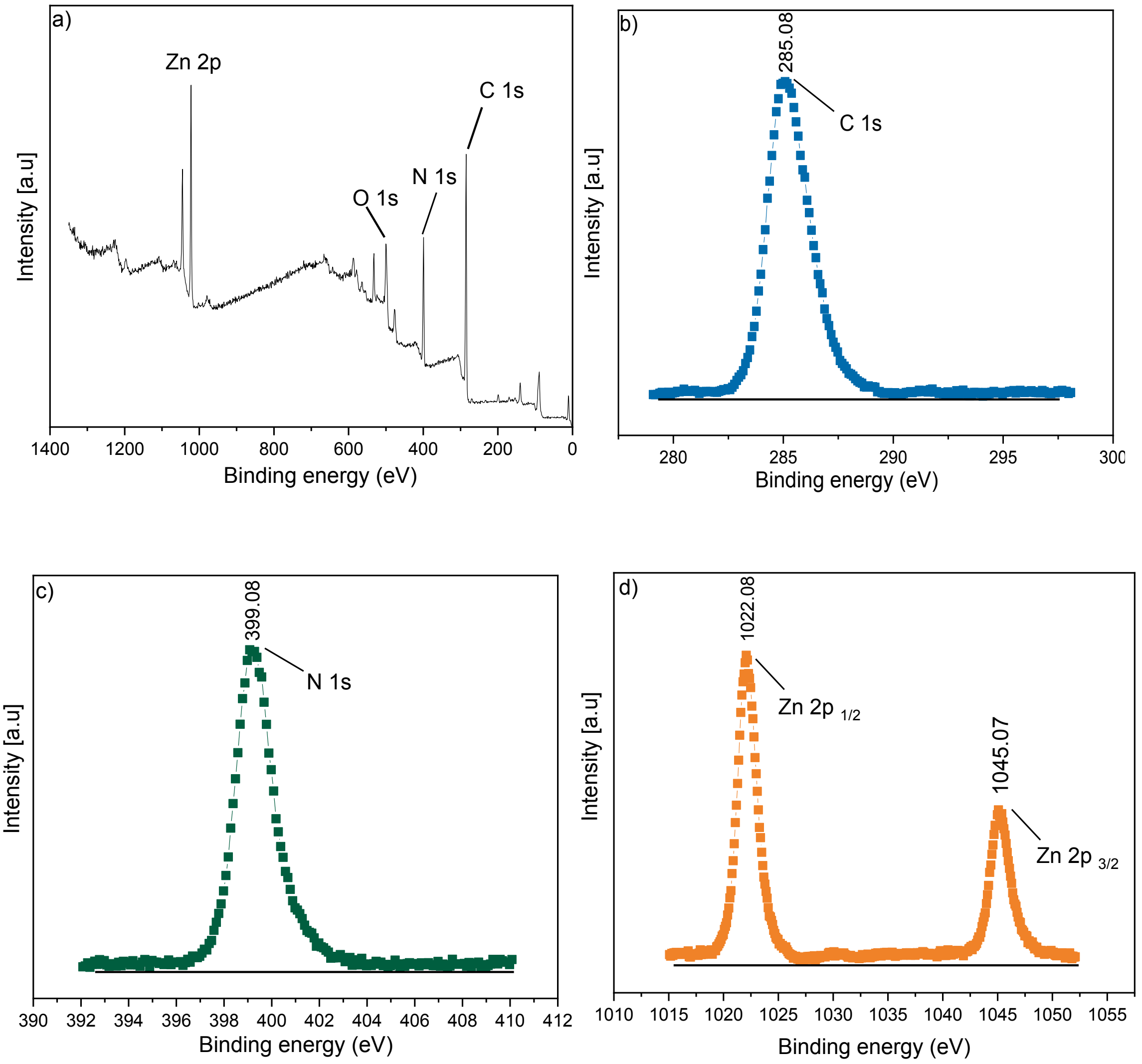

Figure S8.XPS survey (a), C 1s (b), N 1s (c), Zn 2p (d) of Mn/ZIF-8

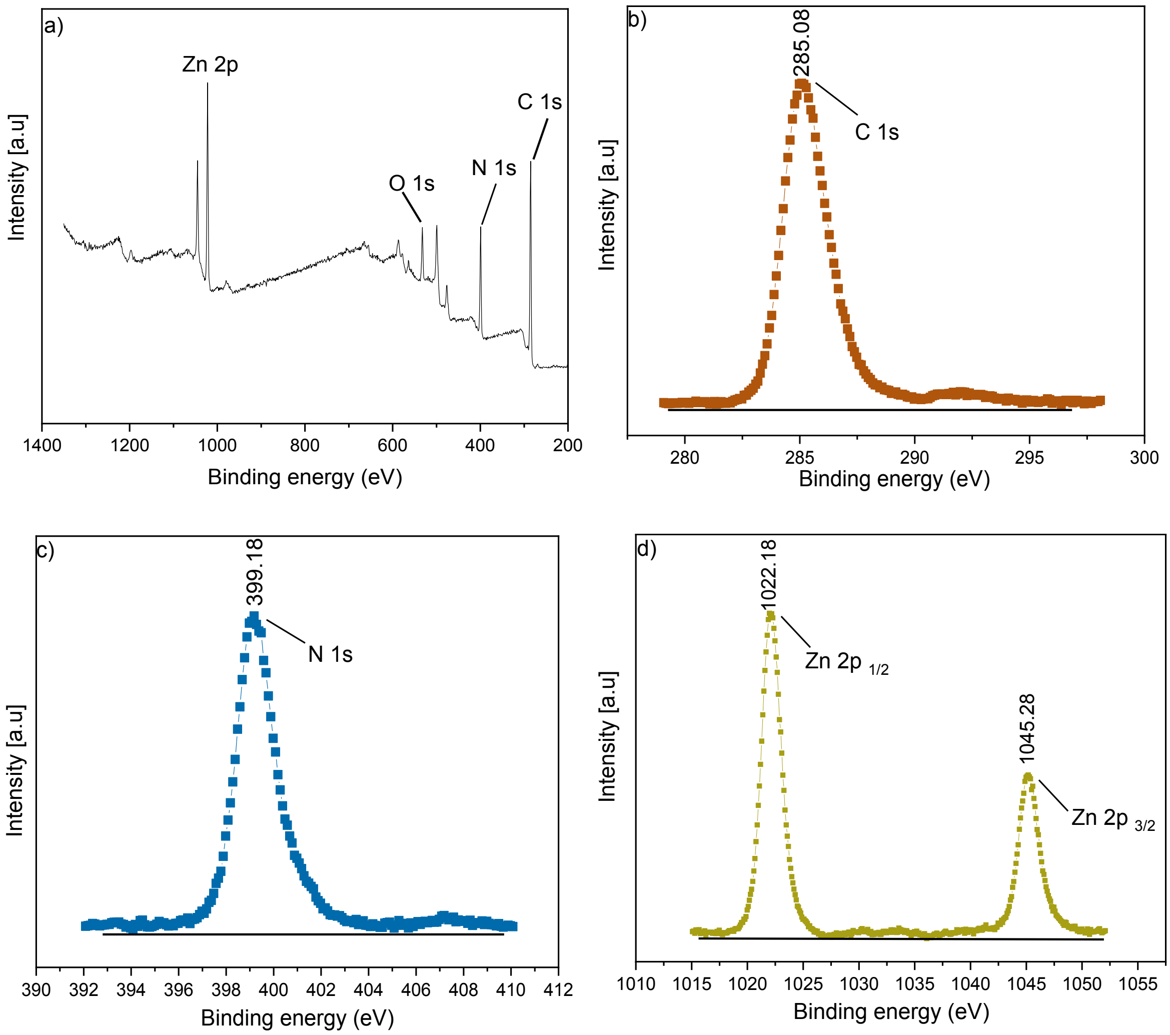

Figure S9.XPS survey (a), C 1s (b), N 1s (c), Zn 2p (d) of Cu-Mn/ZIF-8 (R2)

Table S3. Peak maximum determined from XPS data of samples

Sample	Elements	Binding energy (eV)
ZIF-8	N 1s	399.68
	C 1s	285.33
	Zn 2p $_{1/2}$	1022.48
	Zn 2p $_{3/2}$	1045.58
Cu/ZIF-8	N 1s	399.28
	C 1s	285.28
	Zn 2p $_{1/2}$	1021.98
	Zn 2p $_{3/2}$	1045.16
	Cu 2p $_{1/2}$	932.61
	Cu 2p $_{3/2}$	952.79
Mn/ZIF-8	N 1s	399.08
	C 1s	285.08
	Zn 2p $_{1/2}$	1022.08
	Zn 2p $_{3/2}$	1045.07
Cu-Mn/ZIF-8	N 1s	399.05
	C 1s	284.78
	Zn 2p $_{1/2}$	1021.75
	Zn 2p $_{3/2}$	1044.71
	Cu 2p $_{1/2}$	932.61
	Cu 2p $_{3/2}$	952.79
	Mn 2p $_{1/2}$	641.02
	Mn 2p $_{3/2}$	652.75
Cu-Mn/ZIF-8(R2)	N 1s	399.18
	C 1s	285.08
	Zn 2p $_{1/2}$	1022.18
	Zn 2p $_{3/2}$	1045.28

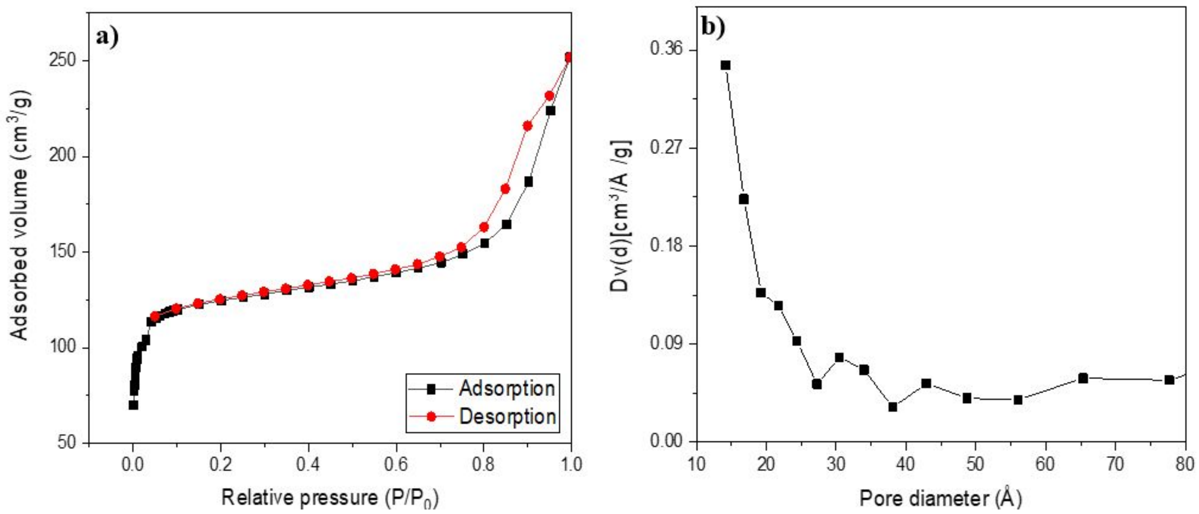

Figure S10. N_2 adsorption-desorption isotherm (a) and pore size distribution (b) of Cu/ZIF-8

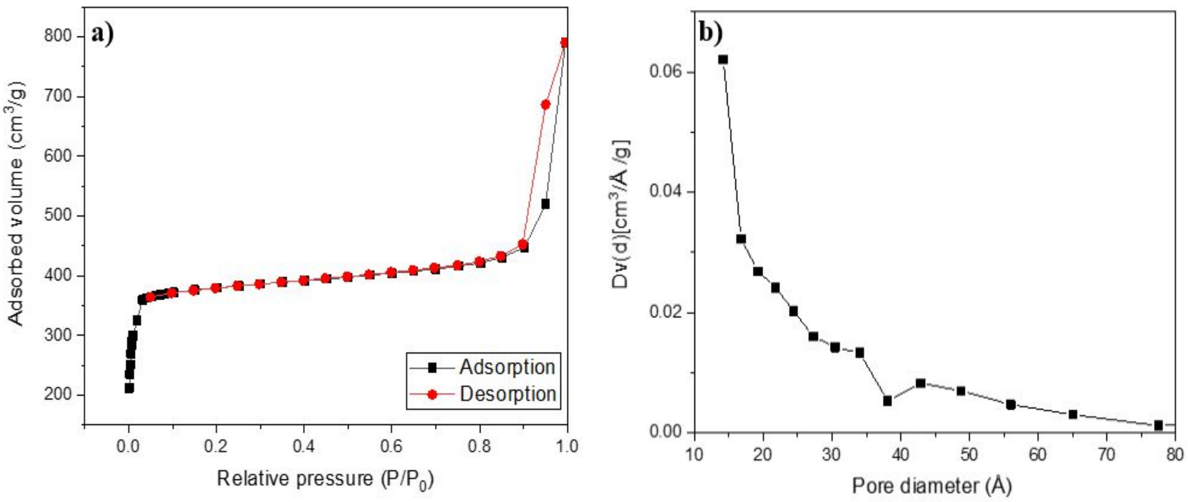

Figure S11. N_2 adsorption-desorption isotherm (a) and pore size distribution (b) of Mn/ZIF-8.

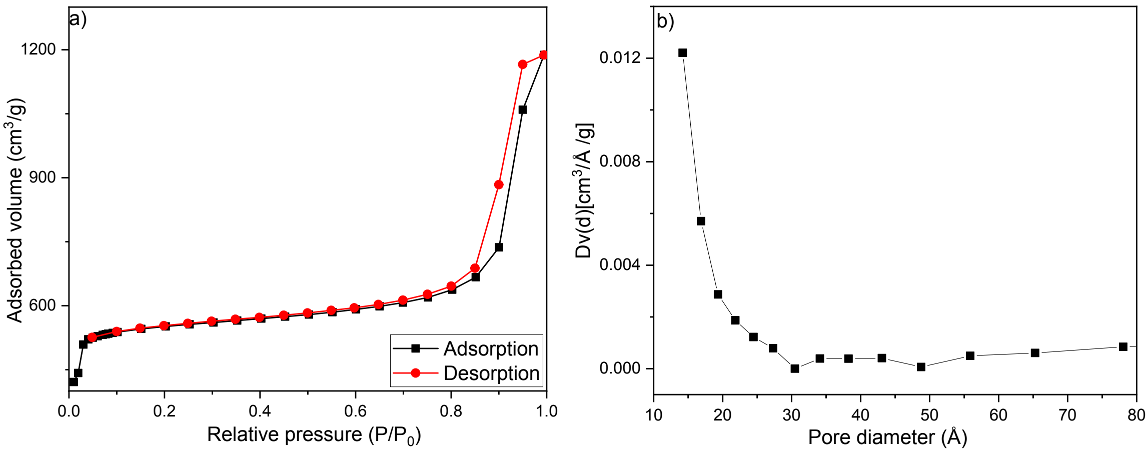

Figure S12 N_2 adsorption-desorption isotherm (a) and pore size distribution (b) of Cu-Mn/ZIF-8

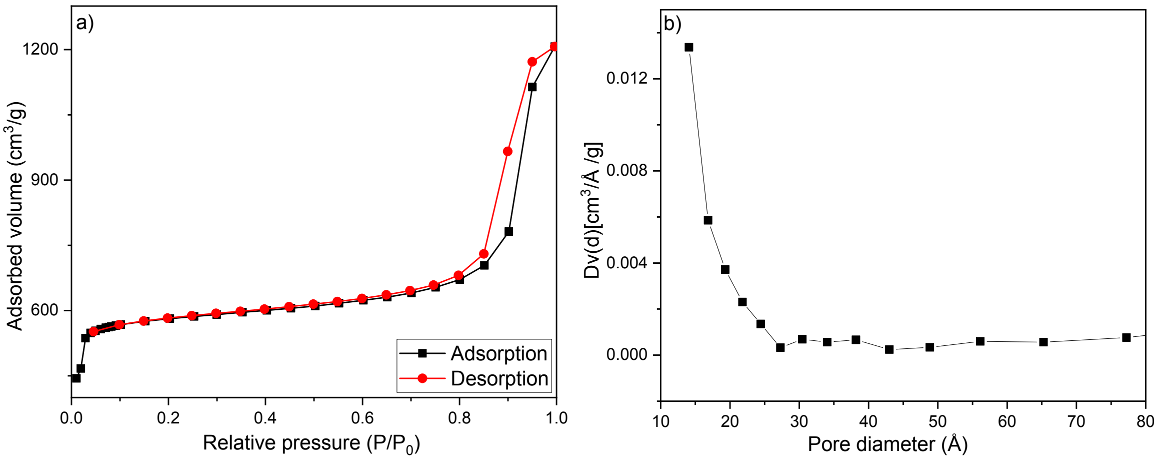

Figure S13. N_2 adsorption-desorption isotherm (a) and pore size distribution (b) of Cu-Mn/ZIF-8 (R1)

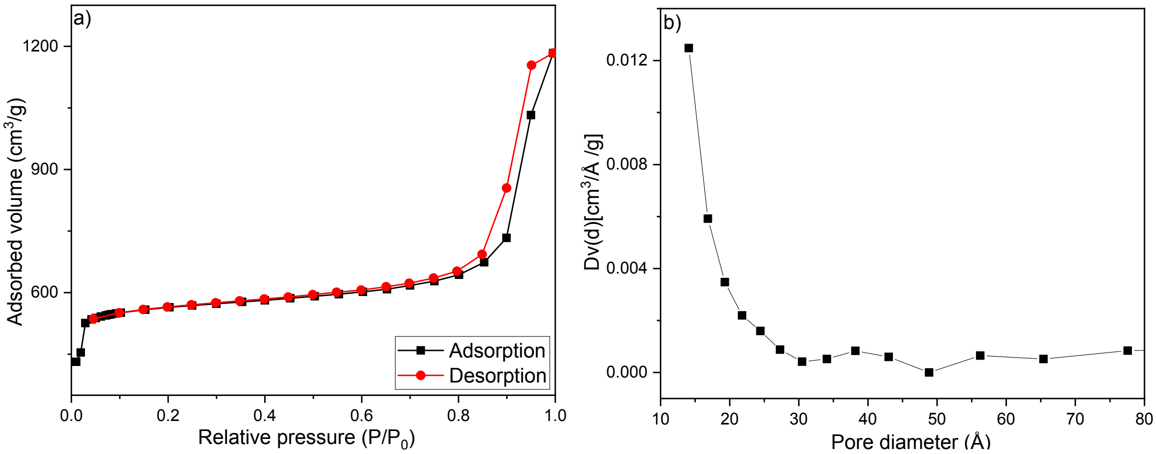

Figure S14. N_2 adsorption-desorption isotherm (a) and pore size distribution (b) of Cu-Mn/ZIF-8 R2

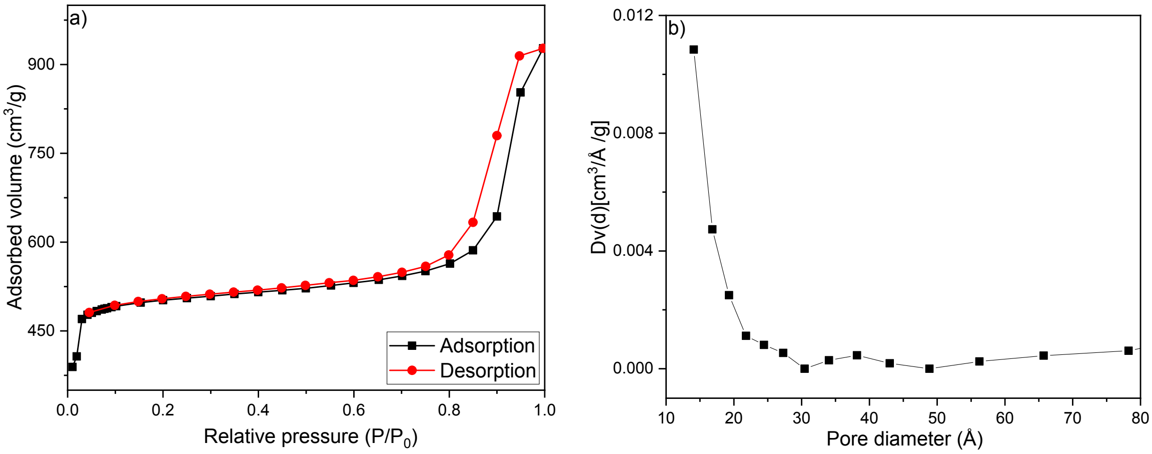

Figure S15. N_2 adsorption-desorption isotherm (a) and pore size distribution (b) of Cu-Mn/ZIF-8 R3

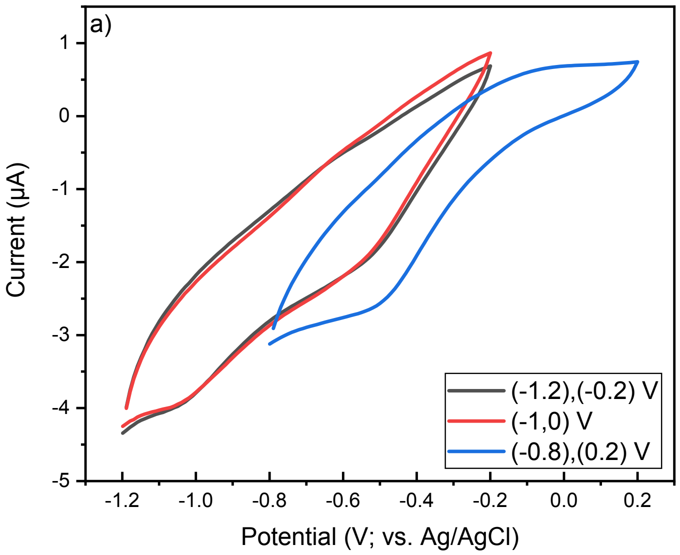

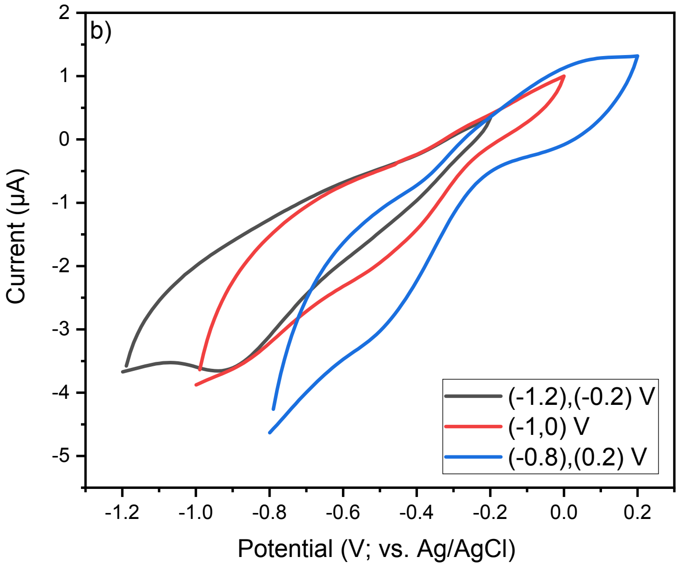

Figure S16**.** CV curves of a) Cu/ZIF-8 b) Mn/ZIF-8 at different potential windows (-1.2 - –0.2 V; -1.0 – 0.0 V; -0.8 – 0.2 V) at 5 mV/s

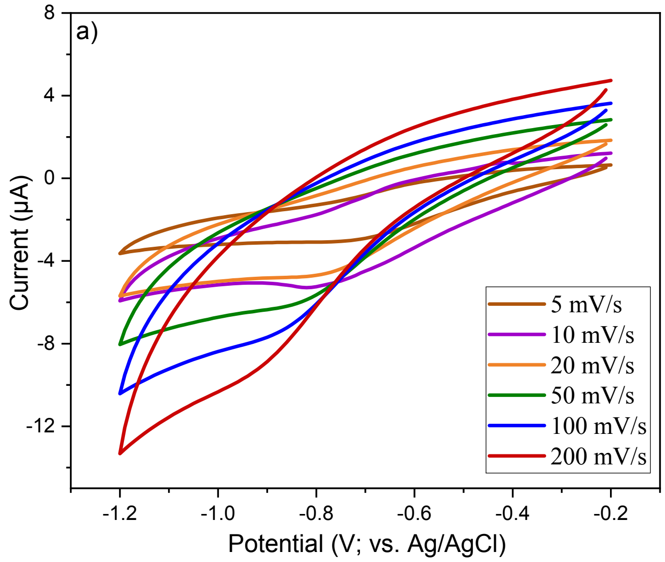
a)
Current (µA)
Potential (V; vs. Ag/AgCl)
5 mV/s
10 mV/s
20 mV/s
50 mV/s
100 mV/s
200 mV/s

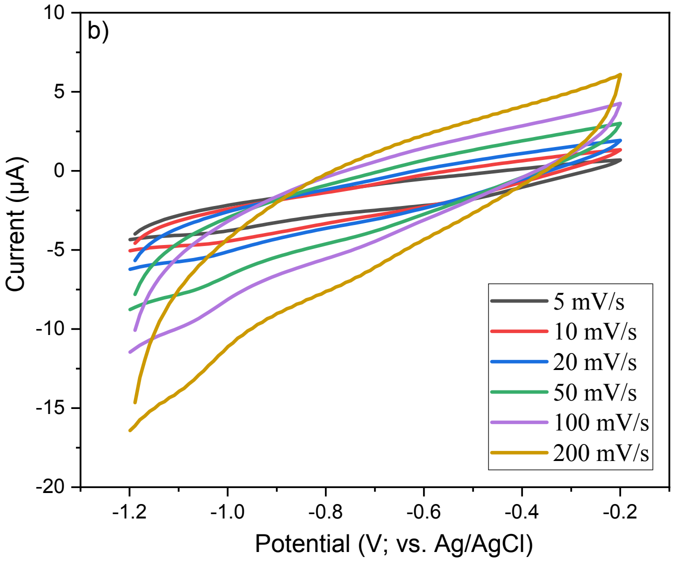
b)
Current (µA)
Potential (V; vs. Ag/AgCl)
5 mV/s
10 mV/s
20 mV/s
50 mV/s
100 mV/s
200 mV/s

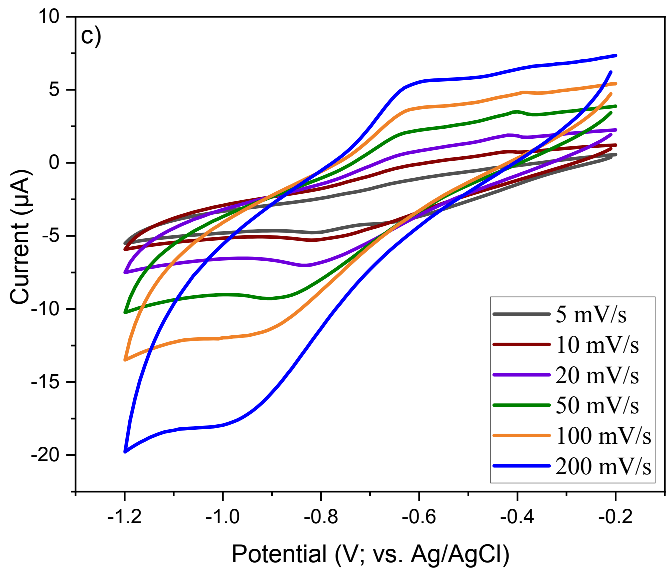
c)
Current (μA)
Potential (V; vs. Ag/AgCl)
5 mV/s
10 mV/s
20 mV/s
50 mV/s
100 mV/s
200 mV/s

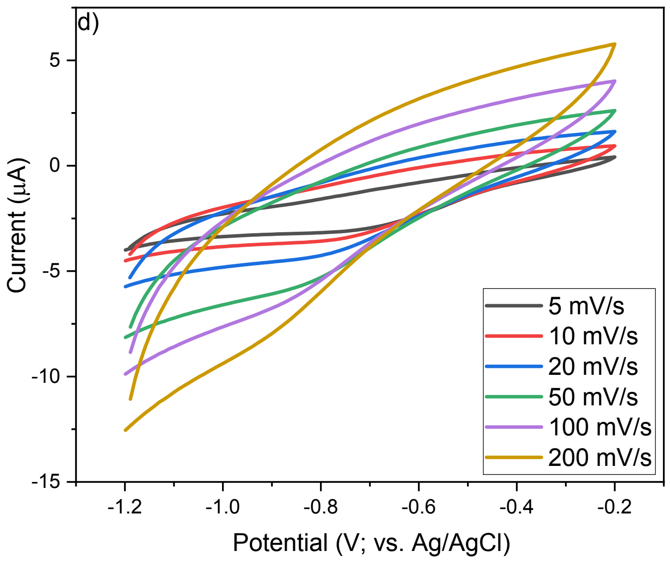
d)
Current (μA)
Potential (V; vs. Ag/AgCl)
5 mV/s
10 mV/s
20 mV/s
50 mV/s
100 mV/s
200 mV/s

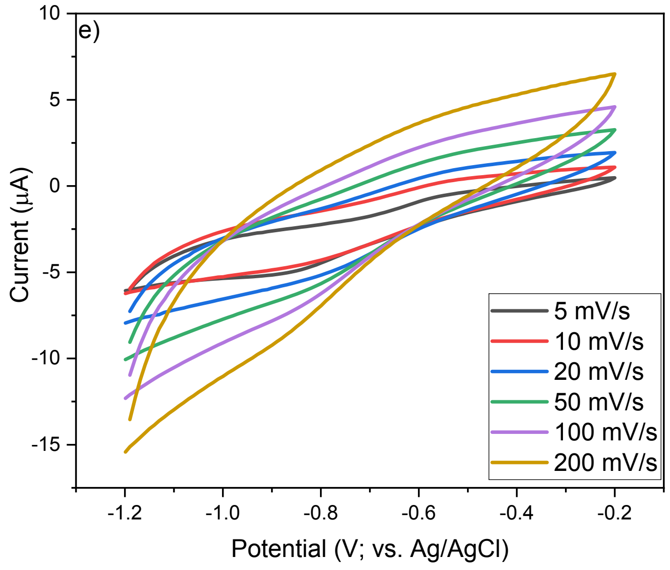

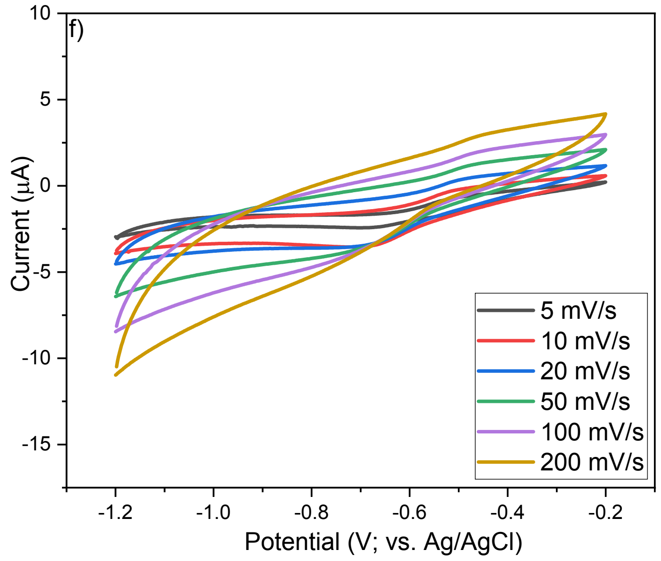

Figure S17.CV curves of a) ZIF-8 b) Cu/ZIF-8 c) Mn/ZIF-8 d) Cu-Mn/ZIF-8 R1 e) Cu-Mn/ZIF-8 R2 f) Cu-Mn/ZIF-8 R3 at different scan rates (5-200 mV/s)
